# Structure Characterization of Four New Sesquiterpene Pyridine Alkaloids from *Tripterygium wilfordii* Hook. f. and Anti-Inflammatory Activity Evaluations

**DOI:** 10.3390/molecules29225284

**Published:** 2024-11-08

**Authors:** Yong-Jian Wang, Jian-Gong Yan, Zhong-Mou Zhang, Qiu-Fang Fang, Ya-Dan Wang, Shuang-Cheng Ma

**Affiliations:** 1 School of Pharmaceutical, Hebei University of Chinese Medicine, Shijiazhuang 050091, China; wyjplh991120@163.com; 2 National Institutes for Food and Drug Control, Beijing 102629, China; yanjiangong@nifdc.org.cn; 3 School of Traditional Chinese Medicine, Beijing University of Chinese Medicine, Beijing 102488, China; zzmsmx@163.com; 4 Faculty of Functional Food and Wine, Shenyang Pharmaceutical University, Shenyang 110016, China; fqf0820@163.com; 5 State Key Laboratory of Drug Regulatory Science, Beijing 100050, China; 6 Chinese Pharmacopoeia Commission, Beijing 100061, China

**Keywords:** *Tripterygium wilfordii*, sesquiterpene pyridine alkaloids (SPAs), anti-inflammatory activity

## Abstract

Sesquiterpene pyridine alkaloids (SPAs), as a main class of components in *Tripterygium wilfordii* Hook. f., possess a variety of bioactivities, such as immunosuppressive, insecticidal, and anti-tumor activities. SPAs can be structurally classed into four subtypes: wilfordate-, evoninate-, iso-wilfordate-, and iso-evoninate types. Our previous study unveiled ten new wilfordate-type SPAs, named wilfordatine A–J, isolated from the roots of *Tripterygium wilfordii* Hook. f., several of which exhibited significant immunosuppressive activities. As an extension and augmentation of the previous findings, we have now isolated one new iso-wilfordate-type SPA, wilfordatine K (**1**), alongside three new iso-evoninate-type SPAs, wilfordatines L–N (**3**–**5**), and six known analogs. Their structures were characterized by the extensive use of 1D and 2D NMR spectroscopic analysis, as well as HRMS data. Interestingly, compounds **4** and **6** were found to exhibit potent inhibitory effects on the nuclear factor-kappa B (NF-κB) pathway in lipopolysaccharide (LPS)-induced HEK293/NF-κB-Luc cells, with IC_50_ values of 1.64 μM and 9.05 μM, respectively. Notably, these two compounds had no influence on the cell viability at a concentration of 100 μM. Consequently, they hold significant promise as potential anti-inflammatory candidates for further exploration and development.

## 1. Introduction

*Tripterygium wilfordii* Hook. f., a member of the Celastraceae family [1,2,3], is widely distributed across various regions in China, including Hunan, Zhejiang, and Fujian provinces. It has been clinically used to treat rheumatoid arthritis, systemic lupus erythematosus, and other autoimmune diseases over a long history [4,5,6]. It mainly contains diterpenes, triterpenes, and sesquiterpene pyridine alkaloids (SPAs) [7,8], among which SPAs are the most abundant.

SPAs were characterized by a macrocyclic diacetone skeleton consisting of a polyoxygenated dihydroagarofuran sesquiterpene cone and a pyridine dicarboxylic acid unit, which can be classified into four subtypes: wilfordate (W), evoninate (E), iso-wilfordate (IW), and iso-evoninate (IE) types [9]. Since the discovery of the first SPA by Kuo et al. in 1989 [10], more than 100 SPAs have been reported to date, with the W-type accounting for the most majority, followed by E-type and, to a lesser extent, the IW-type and IE-type [11].

SPAs possess a variety of biological activities, such as anti-inflammatory [12,13], immunosuppressive [14], anti-tumor [15,16], anti-HIV [17,18,19], and insecticidal properties [20,21,22], the first two of which are particularly relevant to the clinical application of *T. wilfordii*, and several related compounds have been reported. For instance, peritassine A, wilfordinine A, and euonine-suppressed nitric oxide generation at 8.2%, 59.0%, and 54.7%, respectively, in lipopolysaccharide (LPS) induced RAW264.7 cells at a concentration of 5 μM [23]. Moreover, tripterygiumine S and 9′-*O*-acetyl-7-deacetoxy-7-oxowilfortrine exhibited nitric oxide inhibitory effects with respective IC_50_ values of 23.80 ± 4.38 μM and 2.59 ± 3.59 μM in the same cells [24]. It is noteworthy that the abovementioned compounds had no influence on the cell viability. Additionally, ebenifoline E-11 (E) and congorinine E-1 (E) demonstrated significant inhibitory effects on the production of cytokines (TNF-α, IL-1β, IL-8, IL-2, IL-4, and IFN-γ) in human peripheral mononuclear cells stimulated by LPS (or phytohemagglutinin) at a concentration of 10 μg/mL, with the inhibitory rates reaching 100% in some cases [25]. It is evident that SPAs represent a new class of potent anti-inflammatory or immunosuppressive agents with low toxicity, which have received extensive attention from scholars.

**Figure 1:**
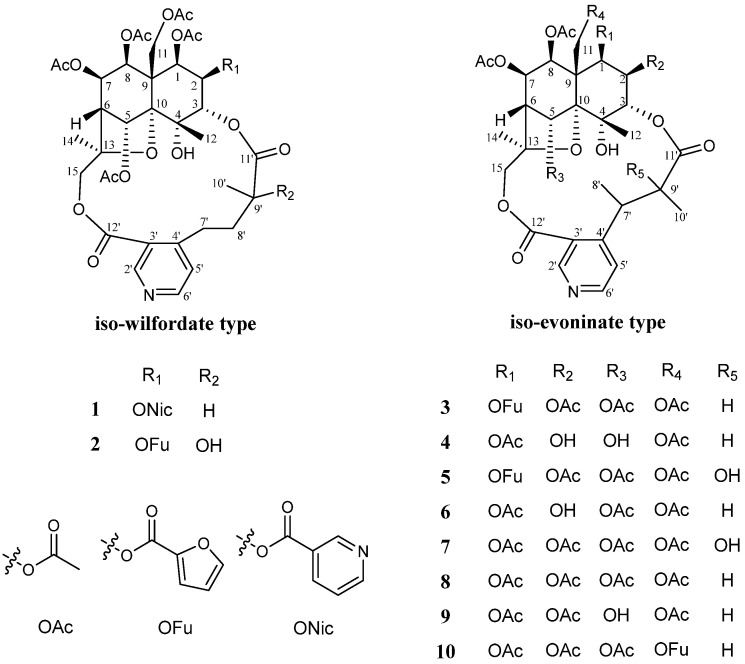
Chemical structures of the isolated SPAs (**1**–**10**).

In a previous study [26], we isolated 20 W-type SPAs from the roots of *T. wilfordii*, including ten new compounds named wilfordatines A–J. Among them, wilfordatine E, tripfordine A, and wilforine showed significant inhibitory activity on the nuclear factor-kappa B (NF-κB) pathway in LPS-induced HEK293/NF-κB-Luc cells, with IC_50_ values of 8.75 μM, 0.74 μM, and 15.66 μM, respectively. As an extension and augmentation of these findings, we have now obtained one new IW-type SPA, wilfordatine K (**1**), alongside three new IE-type SPAs, wilfordatine L–N (**3**–**5**), and six known analogs (Figure 1). The isolation and structural elucidation of these SPAs, as well as the evaluation of their anti-inflammatory activity, will be described in this paper. 

## 2. Results and Discussion

The CHCl_3_-soluble fraction of the ethanol extract of the root of *T. wilfordii* was acid-extracted and alkaline-precipitated to obtain the total alkaloids, which were then subjected to repeated ODS column chromatography and preparative HPLC to yield four new SPAs (**1**, **3**–**5**), along with six known analogs (**2**, **6**–**10**).

**Figure 2:**
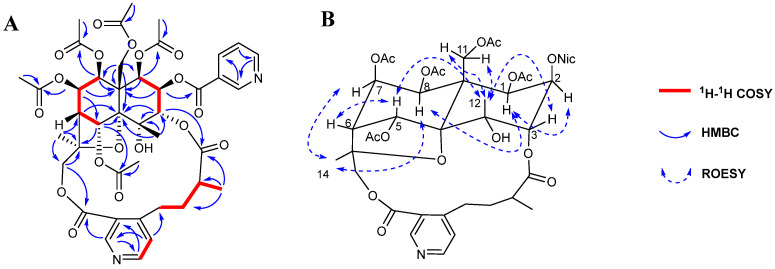
Key 2D NMR correlations of compound **1**: (**A**) The ^1^H-^1^H COSY and HMBC correlations; (**B**) The ROESY correlations.

Compound **1** was isolated as a white amorphous powder. The molecular formula C_42_H_48_O_18_N_2_ was deduced from the quasi-molecular ion [M + H]^+^ at *m*/*z* 869.2991 (calcd 869.2980) in the HRMS data, consistent with 20 degrees of unsaturation (Figure S3). The IR spectrum indicated the presence of hydroxyl (3462 cm^−1^), methyl (2922 cm^−1^), carbonyl (1744 cm^−1^), and ester (1232 cm^−1^) groups (Figure S2), and the UV spectrum suggested the presence of aromatic rings (223 and 264 nm) (Figure S1). The ^1^H NMR data (Table 1, Section 3.4, and Figure S4) of compound **1** showed signals for three methyl groups at δ_H_ 1.64 (3H, s, H-12), 1.71 (3H, s, H-14), and 1.20 (3H, d, *J* = 6.6 Hz, H-10′); six oxygenated methines at *δ*_H_ 6.98 (1H, s, H-5), 5.75 (1H, d, *J* = 3.6 Hz, H-1), 5.55 (1H, t, *J* = 4.2 Hz, H-7), 5.47 (1H, t, *J* = 3.0 Hz, H-2), 5.42 (1H, t, *J* = 4.2 Hz, H-8), and 5.06 (1H, d, *J* = 3.0 Hz, H-3); two sets of oxygenated methylenes at *δ*_H_ 5.47 (1H, d, *J* = 13.2 Hz, H-11a), 4.37 (1H, d, *J* = 13.2 Hz, H-11b), 5.84 (1H, d, *J* = 12.0 Hz, H-15a), and 3.79 (1H, d, *J* = 12.0 Hz, H-15b); two aliphatic methines at *δ*_H_ 2.37 (1H, d, *J* = 3.6 Hz, H-6), and 2.36 (1H, m, H-9′); two sets of aliphatic methylenes at *δ*_H_ 3.90 (1H, m, H-7′a), 2.69 (1H, m, H-7′b), 2.36 (1H, m, H-8′a), and 1.65 (1H, m, H-8′b); 3,4-disubstituted pyridine at *δ*_H_ 9.23 (1H, s, H-2′), 8.69 (1H, d, *J* = 5.4 Hz, H-6′), and 7.27 (1H, overlapped, H-5′); five acetyloxy groups at *δ*_H_ 2.25(3H, s, 11-OAc), 2.19 (3H, s, 7-OAc), 2.18 (3H, s, 5-OAc), 1.96 (3H, s, 8-OAc), and 1.85 (3H, s, 1-OAc); as well as a nicotinoyloxy group at *δ*_H_ 9.31 (1H, d, *J* = 1.8 Hz, 2-ONic-2), 8.37 (1H, d, *J* = 7.8 Hz, 2-ONic-4), 7.48 (1H, dd, *J* = 7.8, 4.8 Hz, 2-ONic-5), and 8.85 (1H, d, *J* = 4.8 Hz, 2-ONic-6). The ^13^C-NMR spectroscopic data (Table 2, Section 3.4, and Figure S5) confirmed the presence of the aforementioned groups, in addition to showing three oxygenated quaternary carbon signals at *δ*_C_ 69.8 (C-4), 93.8 (C-10), and 84.7 (C-13), one aliphatic quaternary carbon signal at *δ*_C_ 52.1 (C-9), and seven ester carbonyl signals at *δ*_C_ 170.4 (11-OAc), 170.1 (7-OAc), 169.9 (5-OAc), 169.3 (1-OAc), 169.0 (8-OAc), 165.8 (2-ONic), 174.6 (C-11′), and 166.7 (C-12′). The ^1^H-^1^H COSY cross signals for H-1/H-2/H-3 and H-5/H-6/H-7/H-8 spin systems and the HMBC correlations for H-1/C-9; H-2/C-9; H-3/C-4, C-10; H-5/C-10, C-13; H-6/C-10; H-7/C-5, C-9; H-8/C-9; H-11/C-1, C-8, C-9, C-10; H-12/C-3, C-4, C-10; H-14/C-6, C-13, C-15; and H-15/C-13 (Figure 2A, Figures S6 and S8) suggested the presence of a polyoxygenated dihydroagarofuran sesquiterpene cone unit. In addition, the ^1^H-^1^H COSY cross signals for H-7′/H-8′/H-9′/H-10′ and the HMBC correlations for H-5′/C-7′; H-2′/C-12′; and H-10′/C-11′ (Figure 2A, Figures S6 and S8), suggested the presence of a 3-carboxyl-α-methyl-4 -pyridinebutanoic acid unit. The linkage of the above two units was via C-3-O-C-11′ and C-15-O-C-12′, as deduced from the key HMBC correlations for H-3/C-11′ and H-15/C-12′. Therefore, compound **1** was concluded to be an IW-type SPA with a nicotinoyloxy, a hydroxy, and five acetoxy groups attached. The positions of the ester groups were undoubtedly assigned by the HMBC correlations for H-11/δ_C_ 170.4 (11-OAc); H-7/δ_C_ 170.1 (7-OAc); H-5/δ_C_ 169.9 (5-OAc); H-1/δ_C_ 169.3 (1-OAc); H-8/δ_C_ 169.0 (8-OAc); and H-2/δ_C_ 165.8 (2-ONic).

The relative configuration of compound **1** was established by the ROESY experiment and coupling constant analysis. The ROESY correlations for H-1/H-8; H-1/H-14; and H-8/H-14 located these protons on the same face as the dihydroagarofuran skeleton (α-orientation) (Figure 2B and Figure S9). Similarly, the correlations for H-3/H-12; H-5/H-12; and H-11/H-12 were located on the other side of the dihydroagarofuran skeleton (β-orientation) (Figure 2B and Figure S9). The coupling constants between H-1 and H-2 (*J*_1,2_ = 3.6 Hz), H-7 and H-8 (*J*_7,8_ = 6.0 Hz) indicated that H-2 and H-7 were of the α-orientation.

Therefore, compound **1** was identified as 2β-nicotinoyloxy-1β,7β,8β,11-tetraacetoxy-4α,5α-dihydroxy-3α,15-[2′-methyl-4′-(3″-carboxy-4″-pyridyl) butanoic acid]-dicarbolactone dihydro-β-agarofuran, and was named wilfordatine K.

**Figure 3:**
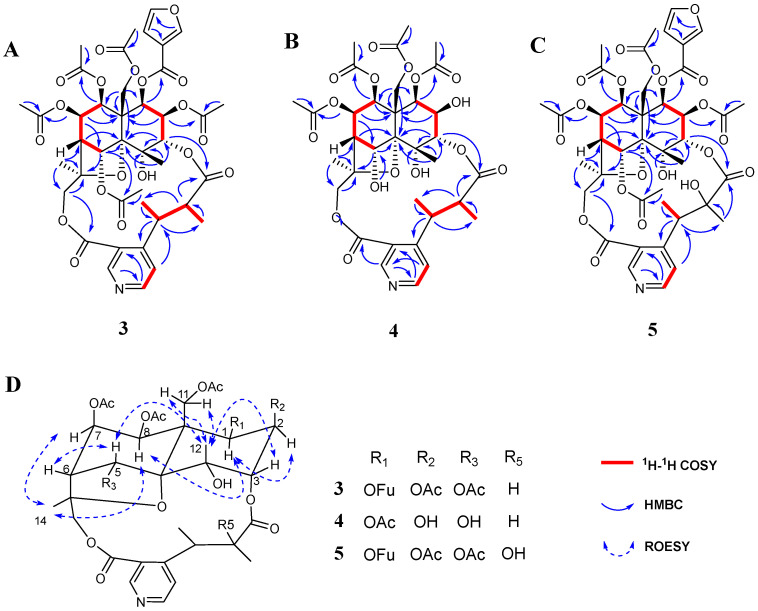
Key 2D NMR correlations of compounds **3**–**5**: (**A**) The ^1^H-^1^H COSY and HMBC correlations of compound **3**; (**B**) The ^1^H-^1^H COSY and HMBC correlations of compound **4**; (**C**) The ^1^H-^1^H COSY and HMBC correlations of compound **5**; (**D**) The ROESY correlations of compounds **3**–**5**.

Compound **3** was obtained as a white amorphous powder. Its molecular formula was determined as C_41_H_47_O_19_N on the basis of the quasi-molecular ion [M + H]^+^ at *m*/*z* 858.2820 (calcd 858.2821) in the HRMS data, consistent with 19 degrees of unsaturation (Figure S12). A comprehensive analysis of compound 3’s IR, UV, and NMR data (Table 1 and Table 2, Section 3.4, and Figures S10, S11, S13 and S14) deduced it to be an SPA. The ^1^H- and ^13^C-NMR data of **3** were similar to those of **1**, with two major differences. First, there were additional furanoyloxy group signals observed in **3** at *δ*_H_ 7.88 (1H, d, *J* = 0.6 Hz, 1-OFu-2), 6.58 (1H, t, *J* = 1.2 Hz, 1-OFu-4), and 7.40 (1H, t, *J* = 1.8 Hz, 1-OFu-5), replacing the nicotinoyloxy group signals in **1**. More significantly, **3** possessed a quite different pyridine dicarboxylic acid moiety from **1**, consisting of a 3, 4-disubstituted pyridine at δ_H_ 9.00 (1H, s, H-2′), 8.72 (1H, d, H-6′), and 7.36 (1H, d, H-5′); two methyl groups at δ_H_ 1.37 (3H, d, *J* = 7.2 Hz, H-8′), and 1.09 (3H, d, *J* = 7.2 Hz, H-10′); and two aliphatic methines at δ_H_ 4.71 (1H, q, *J* = 7.2 Hz, H-7′), and 2.46 (1H, q, *J* = 7.2 Hz, H-9′). It can be seen as a 3-carboxyl-α,β-dimethyl-4-pyridinepropanic acid unit by the ^1^H-^1^H COSY cross signals for H-8′/H-7′/H-9′/H-10′ and the HMBC correlations for H-7′/C-4′; H-8′/C-4′; H-5′/C-7′; H-2′/C-12′; and H-9′/C-11′ (Figure 3A and Figures S15 and S17). The macrocyclic diacetone skeleton was also established by the linkage of C-3-O-C-11′ and C-15-O-C-12′, as evidenced by the key HMBC correlations for H-3/C-11′ and H-15/C-12′ (Figure 3A and Figure S17). Therefore, **3** was concluded to be an IE-type SPA with five acetoxy groups, a hydroxy group, and a furanoyloxy group attached. The positions of the ester groups were assigned by the HMBC correlations for H-11/δ_C_ 170.3 (11-OAc); H-7/δ_C_ 169.9 (7-OAc); H-5/δ_C_ 169.9 (5-OAc); H-8/δ_C_ 168.9.3 (8-OAc); H-2/δ_C_ 168.3 (2-OAc); and H-1/δ_C_ 160.8 (1-OFu).

The relative configuration of the dihydroagarofuran sesquiterpene cone in compound **3** was determined to be identical to that in **1** based on the ROESY correlations for H-8/H-1; H-8/H-14; H-12/H-3; H-12/H-5; and H-12/H-11 (Figure 3D and Figure S18). Thus, the structure of compound **3** was identified to be 1β-furanoyloxy-2β,5α,7β,8β,11-pentaacetoxy-4α-hydroxy-3α,15-[2′,3′-dimethyl-3′-(3″-carboxy-4″-pyridyl) propanic acid] dicarbolactone dihydro-β-agarofuran, and was named wilfordatine L.

Compound **4** was obtained as a white amorphous powder with a molecular formula of C_34_H_43_O_16_N determined by HRMS data (*m*/*z* 722.2645 [M + H]^+^, calcd 722.2660), consistent with 14 degrees of unsaturation (Figure S21). The ^1^H- and ^13^C-NMR data (Table 1 and Table 2, Section 3.4, and Figures S22 and S23) of compound **4** were comparable to those of **3**, except for the absence of an acetyloxy group and a set of 3-furanoyloxy group signals. It is worth noting that compound **4** showed upfield shifts for H-2 (δ_H_ 4.10) and H-5 (δ_H_ 5.45) in contrast to **3** (δ_H_ 5.28 for H-2, δ_H_ 7.07 for H-5), suggesting that the ester groups at C-2 and C-5 in compound **3** are replaced by hydroxy groups in compound **4**. By analyzing the HMBC spectrum, the four acetoxy groups and the 3-furanoyloxy group were assigned to C-1, C-7, C-8, C-11, and C-2, respectively (Figure 3B and Figure S26). The relative configuration of compound **4** was determined to be the same as that of **3** by the ROESY experiment (Figure 3D and Figure S27). Thus, the structure of compound **4** was identified to be 1β,7β,8β,11-tetraacetoxy-1β,4α,5α-trihydroxy-3α,15-[2′,3′-dimethyl-3′-(3″-carboxy-4″-pyridyl) propanic acid] dicarbolactone dihydro-β-agarofuran, and was named wilfordatine M.

Compound **5** was isolated as a white amorphous powder with a molecular formula of C_41_H_47_O_20_N, as supported by HRMS data (*m*/*z* 874.2772 [M + H]^+^, calcd 874.2770), consistent with 19 degrees of unsaturation (Figure S30). A comparison of the ^1^H- and ^13^C-NMR data (Table 1 and Table 2, Section 3.4, and Figures S31 and S32) between compounds **5** and **3** revealed that compound **5** has the same skeleton and substituent groups as **3**, with the main difference being the pyridine dicarboxylic acid moiety. In compound **5**, the proton signal of H-10′ was observed as a singlet, in contrast to the doublet (*J* = 7.2 Hz) in **3**. Furthermore, compound **5** showed a downfield shift for C-9′ (*δ*_C_ 76.7) relative to **3** (*δ*_C_ 45.7), which, combined with the molecular formula, suggested that C-9′ in compound **5** was substituted by a hydroxy group. The six ester groups in compound **5** were assigned to the same locations as **3** by the HMBC spectrum (Figure 3C and Figure S35). Thus, the structure of compound **5** was identified to be 1β-furanoyloxy-2β,5α,7β,8β,11-pentaacetoxy-4α-hydroxy-3α,15-[2′-hydroxy-2′,3′-dimethyl-3′-(3″-carboxy-4″-pyridyl) propanic acid] dicarbolactone dihydro-β-agarofuran, and was named wilfordatine N. 

In addition to the four new SPAs mentioned above, six known analogs were also isolated from *T. wilfordii* and identified as wilfordinine H (**2**) [27], wilfordinine A (**6**) [18], wilfordinine B (**7**) [18], peritassine A (**8**) [28], tripfordine C (**9**) [19], and hypoglaunine D (**10**) [29] by comparing their NMR and HRMS data (Figures S37–S54) with the literature values. 

The inhibitory effects of compounds **2**, **4**, and **6**–**8** on the NF-κB pathway were assessed in LPS-induced HEK293/NF-κB-Luc cells at a concentration of 100 μM. As presented in Table 3, the tested compounds exhibited varying levels of NF-κB inhibition; however, none of them impacted cell viability. Notably, compounds **4** and **6**, which inhibited NF-κB by over 45%, were further determined for their respective IC_50_ values to be 1.64 μM and 9.05 μM, respectively (Figures S55 and S56).

## 3. Materials and Methods

### 3.1. General Experimental Procedures

Optical rotations were recorded using a Rudolph Research Analytical Autopol III polarimeter (Hackettstown, NJ, USA). UV spectra were acquired on a Shmadzu UV-2700 UV–visible spectrophotometer (Kyoto, Japan), and IR spectra were measured on a Nicolet iN10 MX spectrometer (Thermo Fisher Scientific, Waltham, MA, USA). NMR experiments were conducted on a Bruker AV-600 spectrometer (Billerica, MA, USA) in CDCl_3_ with TMS as the internal standard. HRMS data were collected on a Waters Xevo Q-Tof MS spectrometer (Milford, MA, USA). Preparative HPLC separations were performed on a Waters LC Prep 150 System using various columns, such as the Waters XBridge Prep OBD C_18_ column (30 mm × 150 mm, 10 μm), Waters XSelected CSH Prep C_18_ column (19 mm × 250 mm, 5 μm), as well as the YMC-Pack Ph column (10 mm × 250 mm, 5 μm). Neutral alumina (100–200 mesh, Sinopharm Chemical Reagent Co., Ltd., Shanghai, China) and ODS (50 μm, YMC, Kyoto, Japan) were used for column chromatography.

### 3.2. Plant Material

The roots of *Tripterygium wilfordii* Hook. f. were collected in August 2020 from Shaoyang, China. A voucher specimen was identified by Prof. Shuai Kang from the National Institutes for Food and Drug Control (NIFDC) and deposited at the herbarium of NIFDC (No. 10106900006).

### 3.3. Extraction and Isolation

The roots of *T. wilfordii* (50 kg) were pulverized and extracted with 95% ethanol (250 L × 2 h × 3 times) under reflux conditions. The resultant ethanol extract was concentrated under reduced pressure to yield a residue, which was then suspended in water and partitioned using CHCl_3_ (3 times). A total of 120 g of CHCl_3_ soluble extract was dissolved in EtOAc and partitioned three times with a 5% HCl aqueous solution. Then, ammonium hydroxide was added to the HCl aqueous layer to adjust the pH to 8~9. After filtration, the residue was redissolved in EtOAc and subjected to chromatography on a neutral alumina column, eluting with EtOAc. After the recovery of EtOAc by evaporation and drying, 21.36 g of the total alkaloids was obtained.

The total alkaloids (12.76 g) were subjected to ODS column chromatography eluting with CH_3_OH-H_2_O (35:65 → 100:0) to obtain nine fractions (Fr.1–Fr.9). Fr.2 (587 mg) was isolated by preparative HPLC on a Waters XBridge Prep OBD C_18_ column using acetonitrile–H_2_O (30:70, *v*/*v*) as the mobile phase (15 mL/min) to afford six subfractions (Fr.2-1–Fr.2-6). Fr.2-3 (173 mg) and Fr.2-4 (58 mg) were repeatedly purified on a Waters XSelected CSH Prep C_18_ column using an acetonitrile–0.05% trifluoroacetic acid aqueous solution (8 mL/min) to obtain compounds **4** (6.82 mg), and **6** (17.17 mg), respectively. Fr.2-5 (47 mg) was repeatedly purified on a YMC-Pack Ph column using acetonitrile–water (4 mL/min) to afford compound **7** (17.80 mg). Similarly, Fr.4 (2.56 g), Fr.5 (3.40 g), and Fr.6 (2.74 g) were separated by preparative HPLC on a Waters XBridge Prep OBD C_18_ column using acetonitrile–H_2_O (35:65 for Fr.4, 40:60 for Fr.5, and 45:55 for Fr.6, *v*/*v*) as the mobile phase (15 mL/min) to give several subfractions, which were further purified by semipreparative HPLC on a YMC-Pack Ph column. As a result, compounds **5** (4.78 mg) and **8** (8.71 mg) were obtained from Fr.4; compounds **1** (1.80 mg), **2** (229.80 mg), **3** (4.86mg), and **9** (2.53mg) were obtained from Fr.5; and compound **10** (6.80 mg) was obtained from Fr.6.

### 3.4. Characterization of New Compounds

*Wilfordatine K* (**1**): white amorphous powder; ${\left[\alpha \right]}_{D}^{20}$ +11.11 (*c* 0.05, MeOH); UV λMeOH max(log *ε*): 223 (4.18), 264 (3.67) nm; IR (KBr) υ_max_: 3462, 2922, 2851, 2363, 1744, 1647, 1592, 1371, 1276, 1232, 1188, 1165, 1100, 1048, 1002, 976, 882, 739, 700, 600 cm^−1^; HRMS *m*/*z* 869.2991 [M+H]^+^ (calcd for C_42_H_49_N_2_O_18_, 869.2980); ^1^H-NMR, see Table 1 and 1.85 (3H, s, 1-OAc), 2.18 (3H, s, 5-OAc), 2.19 (3H, s, 7-OAc), 1.96 (3H, s, 8-OAc), 2.25 (3H, s, 11-OAc), 9.31 (1H, d, *J* = 1.8 Hz, 2-ONic-2), 8.37 (1H, d, *J* = 7.8 Hz, 2-ONic-4), 7.48 (1H, dd, *J* = 7.8, 4.8 Hz, 2-ONic-5), 8.85 (1H, d, *J* = 4.8 Hz, 2-ONic-6); ^13^C-NMR data, see Table 2 and 169.3/20.5 (1-OAc), 169.9/21.0 (5-OAc), 170.1/21.6 (7-OAc), 169.0/20.5 (8-OAc), 170.4/21.1 (11-OAc), 151.3 (2-ONic-2), 124.8 (2-ONic-3), 137.3 (2-ONic-4), 123.6 (2-ONic-5), 154.3 (2-ONic-6), 165.8 (2-ONic-7).

*Wilfordatine L* (**3**): white amorphous powder; ${\left[\alpha \right]}_{D}^{20}$ −38.18 (*c* 0.06, MeOH); UV λMeOH max(log *ε*): 227 (4.11), 264 (3.69) nm; IR (KBr) υ_max_: 3497, 2997, 1745, 1589, 1554, 1508, 1371, 1301, 1254, 1231, 1187, 1160, 1142, 1121, 1096, 1076, 1057, 1005, 960, 874, 835, 790, 760, 604 cm^−1^; HRMS *m*/*z* 858.2820 [M+H]^+^ (calcd for C_41_H_48_NO_19_, 858.2821); ^1^H-NMR, see Table 1 and 2.16 (3H, s, 2-OAc), 2.13 (3H, s, 5-OAc), 2.21 (3H, s, 7-OAc), 1.65 (3H, s, 8-OAc), 2.34 (3H, s, 11-OAc), 7.88 (1H, d, *J* = 0.6 Hz, 1-OFu-2), 6.58 (1H, t, *J* = 1.2 Hz, 1-OFu-4), 7.40 (1H, t, *J* = 1.8 Hz, 1-OFu-5); ^13^C-NMR data, see Table 2 and 168.3/20.9 (2-OAc), 169.9/21.0 (5-OAc), 169.9/21.6 (7-OAc), 168.9/20.1 (8-OAc), 170.3/21.4 (11-OAc), 147.8 (1-OFu-2), 118.4 (1-OFu-3), 109.4 (1-OFu-4), 144.1 (1-OFu-5), 160.8 (1-OFu-6).

*Wilfordatine M* (**4**): white amorphous powder; ${\left[\alpha \right]}_{D}^{20}$ −13.95 (*c* 0.06, MeOH); UV λMeOH max(log *ε*): 223 (3.83), 264 (3.46) nm; IR (KBr) υ_max_: 3445, 3408, 2992, 2941, 1748, 1595, 1552, 1459, 1408, 1374, 1239, 1208, 1188, 1156, 1115, 1057, 999, 973, 898, 873, 835, 788, 709, 600 cm^−1^; HRMS *m*/*z* 722.2645 [M + H]^+^ (calcd for C_34_H_44_NO_16_, 722.2660); ^1^H-NMR, see Table 1 and 1.93 (3H, s, 1-OAc), 2.13 (3H, s, 7-OAc), 1.97 (3H, s, 8-OAc), 2.20 (3H, s, 11-OAc), 5.88 (1H, d, *J* = 1.2 Hz, 4-OH); ^13^C-NMR data, see Table 2 and 169.4/20.8 (1-OAc), 170.0/21.0 (7-OAc), 169.1/20.5 (8-OAc), 169.6/21.5 (11-OAc).

*Wilfordatine N* (**5**): white amorphous powder; ${\left[\alpha \right]}_{D}^{20}$ +44.32 (*c* 0.09, MeOH); UV λMeOH max(log *ε*): 220 (3.76), 263 (3.45) nm; IR (KBr) υ_max_: 3566, 3504, 2981, 2939, 1748, 1592, 1372, 1303, 1231, 1158, 1122, 1096, 1077, 1050, 1008, 959, 874, 794, 760, 605 cm^−1^; HRMS *m*/*z* 874.2772 [M + H]^+^ (calcd for C_41_H_48_NO_20_, 874.2770); ^1^H-NMR, see Table 1 and 2.19 (3H, s, 2-OAc), 2.15 (3H, s, 5-OAc), 2.33 (3H, s, 7-OAc), 1.67 (3H, s, 8-OAc), 2.41 (3H, s, 11-OAc), 7.90 (1H, d, *J* = 1.2 Hz, 1-OFu-2), 6.59 (1H, d, *J* = 1.8 Hz, 1-OFu-4), 7.41 (1H, t, *J* = 1.8 Hz, 1-OFu-5); ^13^C-NMR data, see Table 2 and 168.2/20.8 (2-OAc), 169.8/21.0 (5-OAc), 169.5/21.6 (7-OAc), 168.7/20.1 (8-OAc), 170.2/21.4 (11-OAc), 147.9 (1-OFu-2), 118.2 (1-OFu-3), 109.4 (1-OFu-4), 144.2 (1-OFu-5), 160.9 (1-OFu-6).

### 3.5. Bioassays

The effect of the test compounds on the viability of HEK293 cells was determined by the CCK-8 method [26,30,31]. Briefly, HEK293 cells were inoculated into 96-well plates with 5 × 10^3^ cells per well and incubated at 37 °C with 5% CO_2_ for 24 h. The cells were treated with test compounds and incubated for 48 h; then, 20 μL of the CCK-8 reagent was added into each well and incubated for another 2 h. The absorbance at 450 nm was measured with a microplate reader to further calculate the cell viability. All experiments were performed in triplicate.

Anti-inflammation bioassays were carried out using the HEK293/NF-κB-Luc cells, which were produced as the described procedure [26,32,33]. The HEK293/NF-κB-Luc cells were inoculated into 48-well plates and cultured in DMEM supplemented with 10% fetal bovine serum (FBS) for 16 h. Following treatment with the tested compounds, the cells were simulated with 1 μg/mL of LPS for a duration of 24 h. Then, the cells were rinsed twice with phosphate-buffered saline (PBS, pH 7.4) prior to lysing with passive lysis buffer. Then, the inhibitory effects on NF-κB were analyzed using the luciferase assay system according to the manufacturer’s instructions. All the experiments were performed in triplicate.

## 4. Conclusions

In summary, our continued phytochemical investigation of the total alkaloids of the roots of *T. wilfordii* led to the identification of ten SPAs belonging to IW- and IE-types, including four new compounds (**1**, **3**–**5**). Among them, compounds **4** and **6** exhibited potent NF-κB inhibitory activity in LPS-induced HEK293/NF-κB-Luc cells with no cytotoxicity, which holds significant promise as potential anti-inflammatory candidates for further exploration and development.

## Figures and Tables

**Table 1:** ^1^H-NMR data of the skeletons for compounds **1**, **3**–**5** (CDCl_3_, 600 MHz).

Position	*δ*_H_ (*J* in Hz) ^a^			
	1	3	4	5
1	5.75 (1H, d, 3.6)	5.79 (1H, d, 4.2)	5.46 (1H, d, 3.6)	5.84 (1H, d, 3.6)
2	5.47 (1H, t, 3.0)	5.28 (1H, dd, 3.6, 2.4)	4.10 (1H, t, 3.0)	5.39 (1H, dd, 3.6, 2.4)
3	5.06 (1H, d, 3.0)	4.78 (1H, d, 2.4)	4.80 (1H, d, 2.4)	4.75 (1H, d, 2.4)
5	6.98 (1H, s)	7.07 (1H, s)	5.45 (1H, s)	7.07 (1H, s)
6	2.37 (1H, d, 3.6)	2.37 (1H, d, 4.2)	2.44 (1H, d, 3.0)	2.42 (1H, d, 3.6)
7	5.55 (1H, t, 4.2)	5.54 (1H, dd, 6.0, 4.2)	5.51 (1H, dd, 6.0, 4.2)	5.54 (1H, dd, 6.0, 4.2)
8	5.42 (1H, d, 6.0)	5.43 (1H, d, 6.0)	5.34 (1H, d, 6.0)	5.41 (1H, d, 6.0)
11	5.47 (1H, d, 13.2) 4.37 (1H, d, 13.2)	5.25 (1H, d, 13.2) 4.59 (1H, d, 13.2)	5.24 (1H, d, 13.2) 4.64 (1H, d, 13.2)	5.25 (1H, d, 13.2) 4.59 (1H, d, 13.2)
12	1.64 (3H, s)	1.57 (3H, s)	1.86 (3H, d, 1.2)	1.56 (3H, s)
14	1.71 (3H, s)	1.75 (3H, s)	1.67 (3H, s)	1.65 (3H, s)
15	5.84 (1H, d, 12.0) 3.79 (1H, d, 12.0)	6.05 (1H, d, 11.4) 3.70 (1H, d, 11.4)	6.06 (1H, d, 12.0) 3.72 (1H, d, 12.0)	5.11 (1H, d, 11.4) 4.29 (1H, d, 11.4)
2′	9.23 (1H, s)	9.00 (1H, s)	9.04 (1H, br, s)	9.00 (1H, s)
5′	7.27 (1H, overlapped)	7.36 (1H, d, 5.4)	7.37 (1H, br, s)	7.83 (1H, d, 5.4)
6′	8.69 (1H, d, 5.4)	8.72 (1H, d, 5.4)	8.72 (1H, br, s)	8.69 (1H, d, 5.4)
7′	3.90 (1H, m) 2.69 (1H, m)	4.71 (1H, q, 7.2)	4.79 (1H, q, 7.2)	4.25 (1H, q, 7.2)
8′	2.36 (1H, m) 1.65 (1H, m)	1.37(3H, d, 7.2)	1.34 (3H, d, 7.2)	1.19 (3H, d, 7.2)
9′	2.36 (1H, m)	2.46 (1H, q, 7.2)	2.43 (1H, q, 7.2)	
10′	1.20 (3H, d, 6.6)	1.09 (3H, d, 7.2)	1.03 (3H, d, 7.2)	1.39 (3H, s)

^a^ The ^1^H-NMR data of the substituent groups for compounds **1**, **3**–**5** are present in Section 3.4.

**Table 2:** ^13^C-NMR data of the skeleton for compounds **1**, **3**–**5** (CDCl_3_, 150 MHz).

Position	*δ*_C_ ^a^			
	1	3	4	5
1	73.4	73.0	75.4	72.5
2	70.3	69.1	69.4	68.3
3	75.9	75.8	77.3	77.4
4	69.8	70.8	72.5	70.4
5	73.6	73.7	74.3	74.2
6	51.3	50.5	51.8	50.5
7	69.0	68.8	69.1	68.8
8	70.7	71.3	71.0	71.2
9	52.1	52.3	51.4	52.5
10	93.8	94.3	93.2	93.2
11	60.3	59.8	60.7	59.7
12	23.0	22.7	23.4	22.2
13	84.7	84.5	84.5	83.4
14	17.9	18.4	18.6	18.5
15	70.4	70.2	71.1	69.8
2′	152.0	151.0	151.2	151.5
3′	124.4	125.2	123.6	127.4
4′	155.0	156.5	156.4	151.6
5′	126.4	121.5	122.0	123.4
6′	153.4	153.0	152.9	152.6
7′	31.1	33.3	33.0	41.8
8′	38.1	11.3	11.2	17.2
9′	81.8	45.7	45.8	76.7
10′	22.1	10.1	9.4	23.9
11′	174.6	173.6	173.7	175.0
12′	166.7	168.1	168.2	167.6

^a^ The ^13^C-NMR data of the substituent groups for compounds **1** and **3**–**5** are present in Section 3.4.

**Table 3:** NF-κB inhibitory effects of **2**, **4**, and **6**–**8** in LPS-induced HEK293/NF-κB-Luc cells.

Compounds	Cell Viability (%) (*n* = 3)	NF-κB Inhibitory Rates (%) (*n* = 3)	IC_50_ (μM)
**2**	94.25 ± 5.75	33.87 ± 2.06	
**4**	100.00 ± 2.55	56.41 ± 2.17	1.64
**6**	92.38 ± 7.62	48.28 ± 2.84	9.05
**7**	95.01 ± 36.23	32.12 ± 5.70	
**8**	93.90 ± 6.10	19.11 ± 3.20	
Blank control	100.00 ± 3.33		
JSH23 ^a^		74.56 ± 1.83	

^a^ JSH23 was used as positive control for NF-κB inhibition.

## Data Availability

The data presented in this study are available in the article and Supplementary Materials.

## Supplementary Materials

The following supporting information can be downloaded at https://www.mdpi.com/article/10.3390/molecules29225284/s1. Figures S1–S36: 1D-, 2D-NMR, HRMS, UV, and IR spectra for the new compounds; Figures S37–S54: HRMS, ^1^H- and ^13^C-NMR spectra for the known compounds; and Figures S55 and S56: NF-κB inhibitory effect of compounds **2** and **6**.
