# Supplementary material for: Structure Characterization of Four New Sesquiterpene Pyridine Alkaloids from Tripterygium wilfordii Hook. f. and Anti-Inflammatory Activity Evaluations
Source: Molecules. 2024 Nov 8;29(22):5284. doi: 10.3390/molecules29225284 (PMC11596599; doi:10.3390/molecules29225284)
Supplement: Supplementary file 1 [file molecules-29-05284-s001.zip › molecules-3255957-supplementary.pdf]

Structure Characterization of Four New Sesquiterpene Pyridine Alkaloids from Tripterygium wilfordii Hook. f. and Anti-Inflammatory Activity Evaluations

Yong-Jian Wang [1,†], Jian-Gong Yan [2,†], Zhong-Mou Zhang [3], Qiu-Fang Fang [4], Ya-Dan Wang [2,5,*] and Shuang-Cheng Ma [5,6,*]

[1] School of Pharmaceutical, Hebei University of Chinese Medicine, Shijiazhuang 050091, China
[2] National Institutes for Food and Drug Control, Beijing 102629, China
[3] School of Traditional Chinese Medicine, Beijing University of Chinese Medicine, Beijing 102488, China
[4] Faculty of Functional Food and Wine, Shenyang Pharmaceutical University, Shenyang 110016, China
[5] State Key Laboratory of Drug Regulatory Science, Beijing 100050, China
[6] Chinese Pharmacopoeia Commission, Beijing 100061, China
* Correspondence: y.dwang@163.com (Y.-D.W.); masc@chp.org.cn (S.-C.M.)
† These authors contributed equally to this work.

Supplementary information

Table of content

Figure S1. UV spectrum of compound **1** in methanol....4

Figure S2. IR spectrum of compound **1** (KBr disc)....4

Figure S3. HRMS spectrum of compound **1**....5

Figure S4. ^{1}H-NMR spectrum of compound **1** ($CDCl_3$, 600 HMz)....5

Figure S5. ^{13}C-NMR spectrum of compound **1** ($CDCl_3$, 150 HMz)....6

Figure S6. ^{1}H-^{1}H COSY spectrum of compound **1**....6

Figure S7. HSQC spectrum of compound **1**....7

Figure S8. HMBC spectrum of compound **1**....7

Figure S9. ROESY spectrum of compound **1**....8

Figure S10. UV spectrum of compound **3** in methanol....8

Figure S11. IR spectrum of compound **3** (KBr disc)....9

Figure S12. HRMS spectrum of compound **3**....9

Figure S13. ^{1}H-NMR spectrum of compound **3**....10

Figure S14. ^{13}C-NMR spectrum of compound **3**....10

Figure S15. ^{1}H-^{1}H COSY spectrum of compound **3**....11

Figure S16. HSQC spectrum of compound **3**....11

Figure S17. HMBC spectrum of compound **3**....12

Figure S18. ROESY spectrum of compound **3**....12

Figure S19. UV spectrum of compound **4** in methanol....13

Figure S20. IR spectrum of compound **4** (KBr disc)....13

Figure S21. HRMS spectrum of compound **4**....14

Figure S22. ^{1}H-NMR spectrum of compound **4**....14

Figure S23. ^{13}C-NMR spectrum of compound **4**....15

Figure S24. ^{1}H-^{1}H COSY spectrum of compound **4**....15

Figure S25. HSQC spectrum of compound **4**....16

Figure S26. HMBC spectrum of compound **4**....16

Figure S27. ROESY spectrum of compound **4** 17
Figure S28. UV spectrum of compound **5** in methanol......17
Figure S29. IR spectrum of compound **5** (KBr disc) 18
Figure S30. HRMS spectrum of compound **5** 18
Figure S31. ^{1}H-NMR spectrum of compound **5**......19
Figure S32. ^{13}C-NMR spectrum of compound **5**......19
Figure S33. ^{1}H-^{1}H COSY spectrum of compound **5**......20
Figure S34. HSQC spectrum of compound **5**......20
Figure S35. HMBC spectrum of compound **5**......21
Figure S36. ROESY spectrum of compound **5** 21
Figure S37. HRMS spectrum of compound **2** 22
Figure S38. ^{1}H-NMR spectrum of compound **2**......22
Figure S39. ^{13}C-NMR spectrum of compound **2**......23
Figure S40. HRMS spectrum of compound **6** 23
Figure S41. ^{1}H-NMR spectrum of compound **6**......24
Figure S42. ^{13}C-NMR spectrum of compound **6**......24
Figure S43. HRMS spectrum of compound **7** 25
Figure S44. ^{1}H-NMR spectrum of compound **7**......25
Figure S45. ^{13}C-NMR spectrum of compound **7**......26
Figure S46. HRMS spectrum of compound **8** 26
Figure S47. ^{1}H-NMR spectrum of compound **8**......27
Figure S48. ^{13}C-NMR spectrum of compound **8**......27
Figure S49. HRMS spectrum of compound **9** 28
Figure S50. ^{1}H-NMR spectrum of compound **9**......28
Figure S51. ^{13}C-NMR spectrum of compound **9**......29
Figure S52. HRMS spectrum of compound **10** 29
Figure S53. ^{1}H-NMR spectrum of compound **10**......30
Figure S54. ^{13}C-NMR spectrum of compound **10**......30
Figure S55. NF-κB inhibitory effect of compound **2** 31
Figure S56. NF-κB inhibitory effect of compound **6** 31

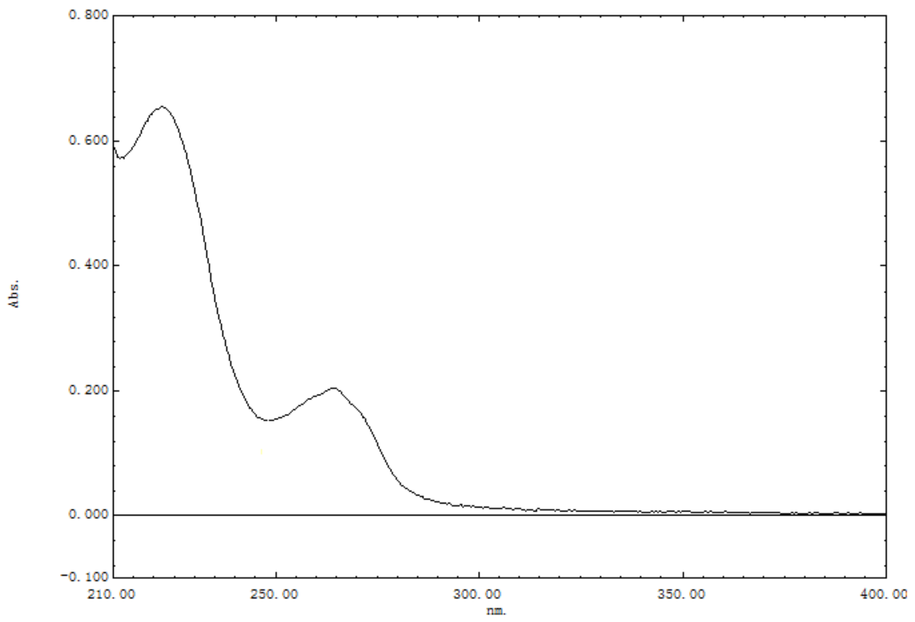

Figure S1. UV spectrum of compound **1** in methanol

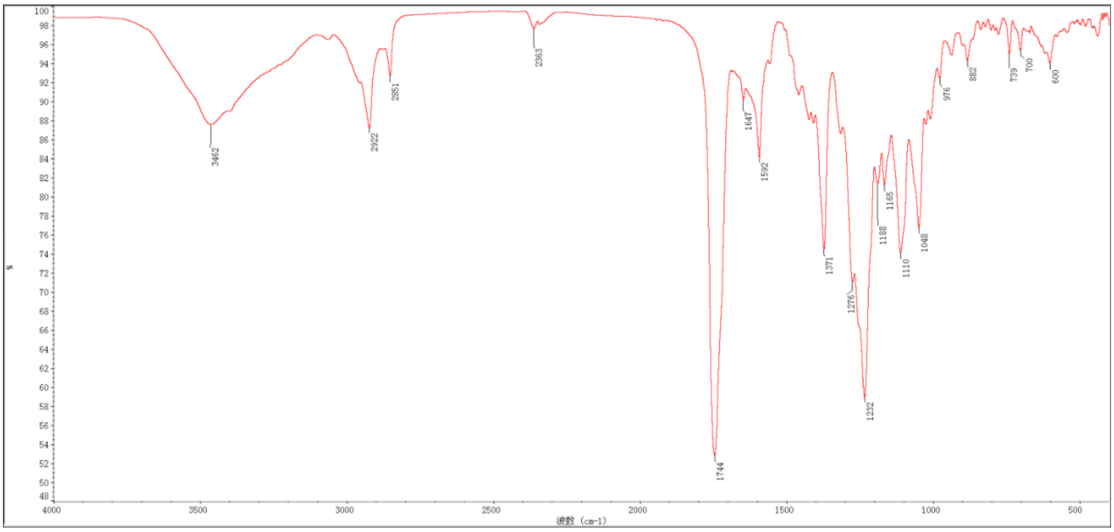

Figure S2. IR spectrum of compound **1** (KBr disc)

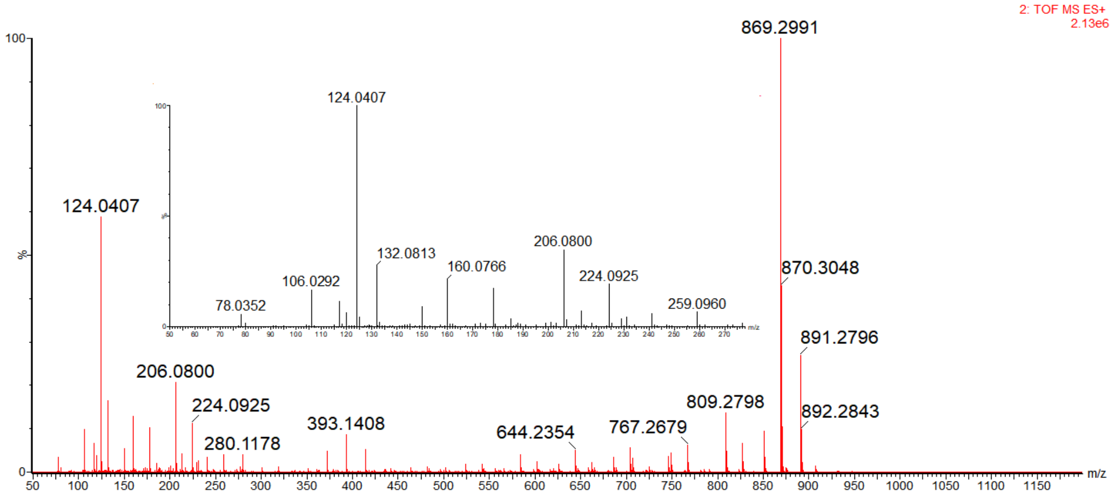

Figure S3. HRMS spectrum of compound **1**

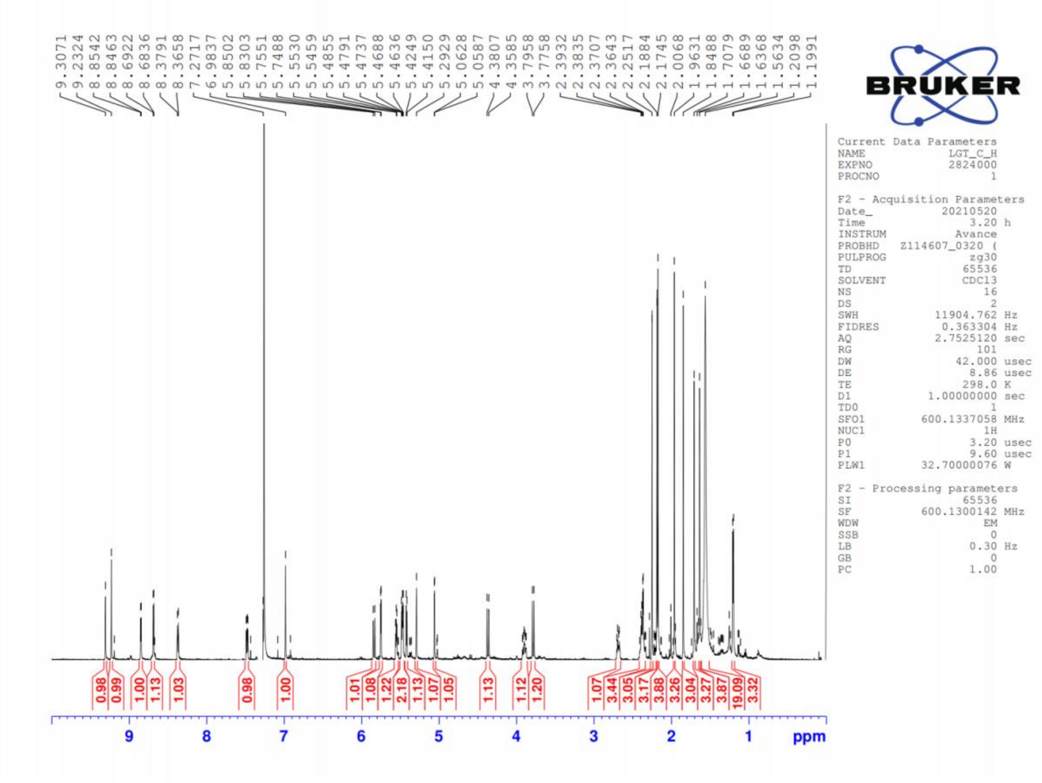

Figure S4. ^1^H-NMR spectrum of compound **1** ($CDCl_3$, 600 HMz)

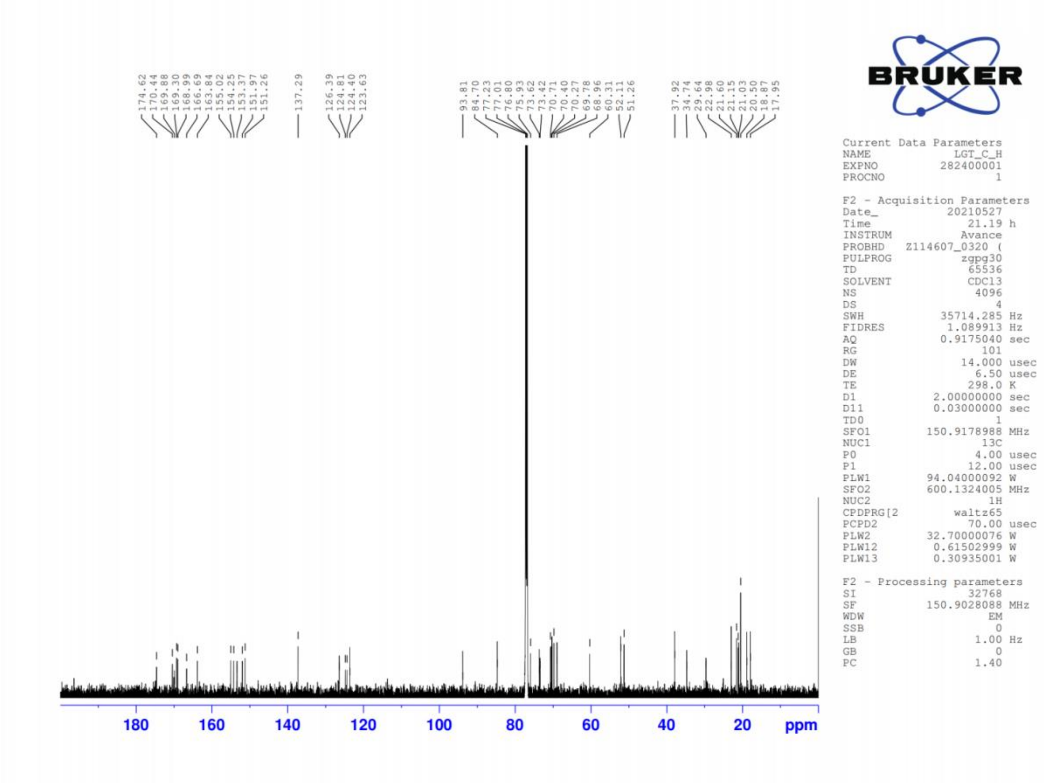

Figure S5. ^{13}C-NMR spectrum of compound **1** ($CDCl_3$, 150 HMz)

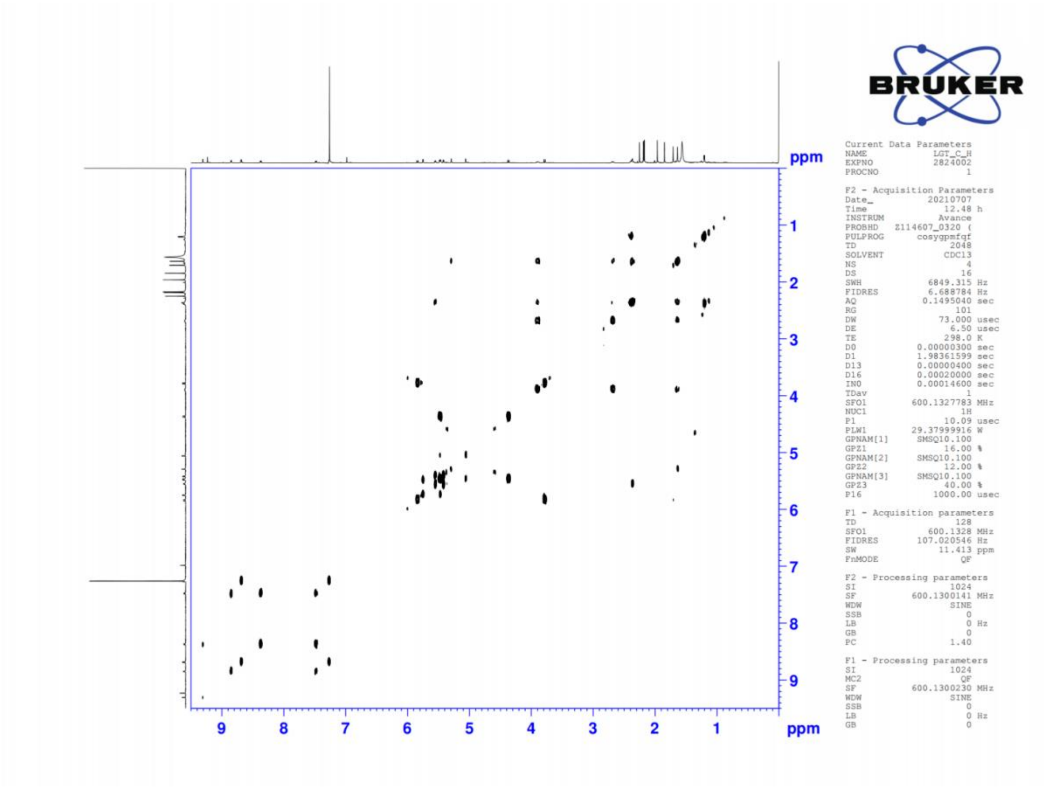

Figure S6. ^{1}H-^{1}H COSY spectrum of compound **1**

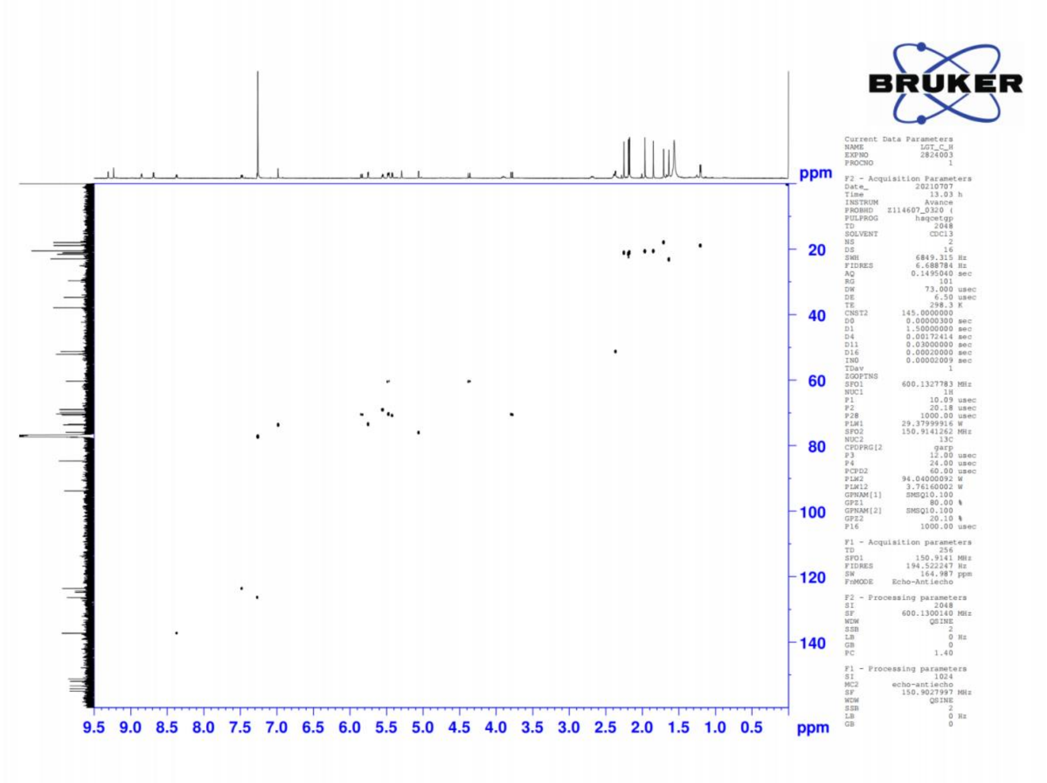

Figure S7. HSQC spectrum of compound **1**

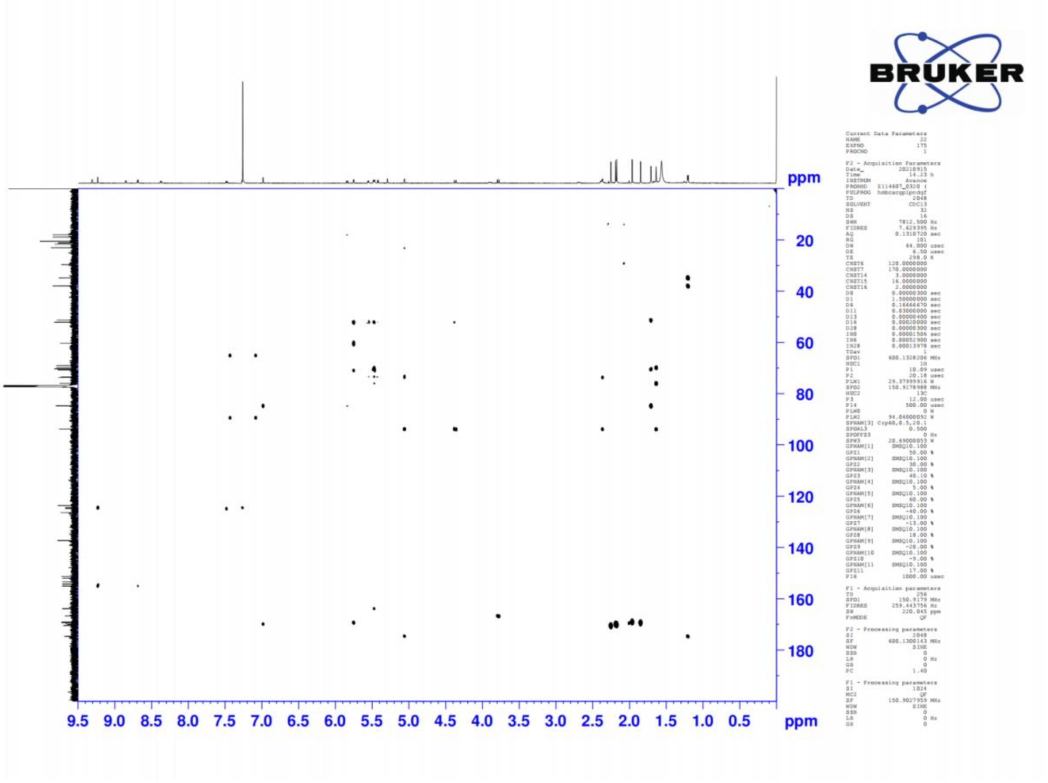

Figure S8. HMBC spectrum of compound **1**

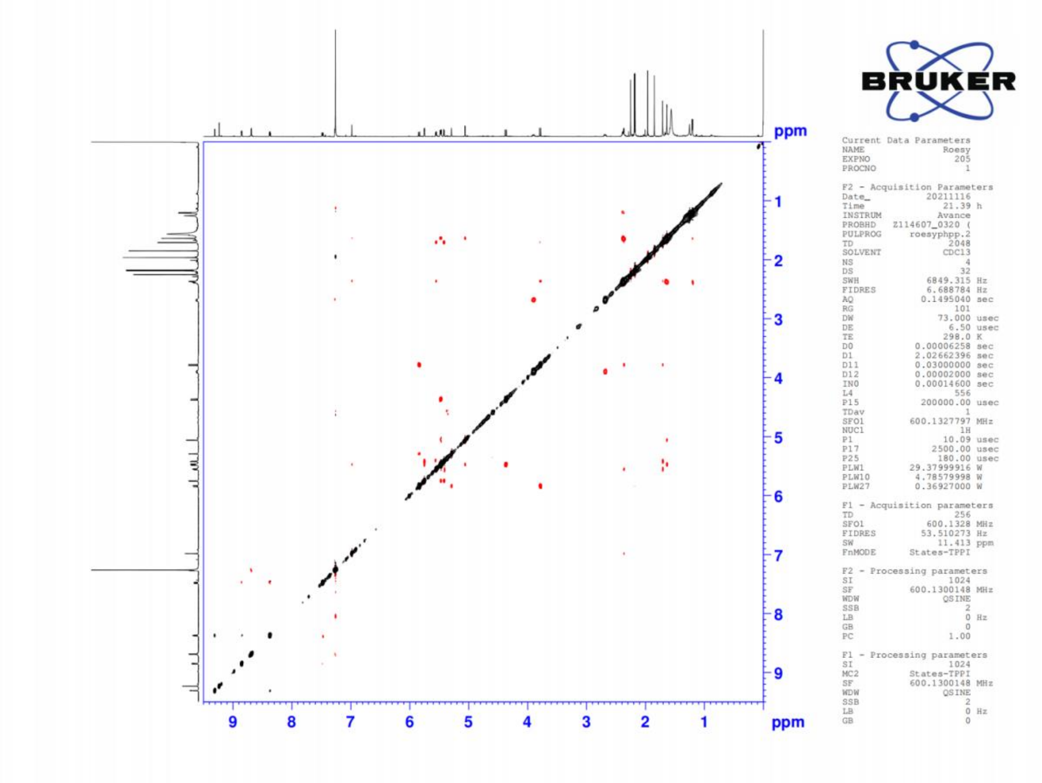

Figure S9. ROESY spectrum of compound **1**

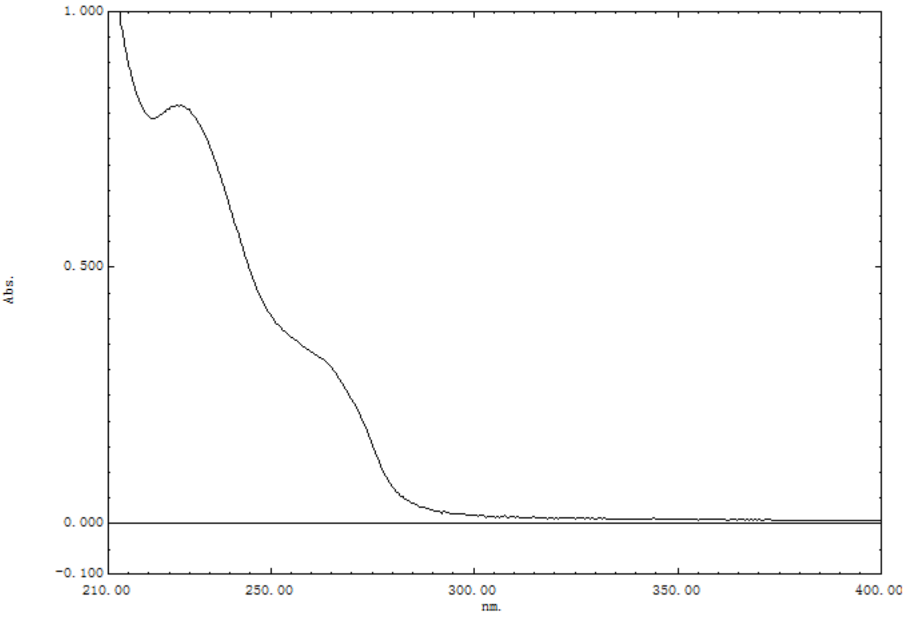

Figure S10. UV spectrum of compound **3** in methanol

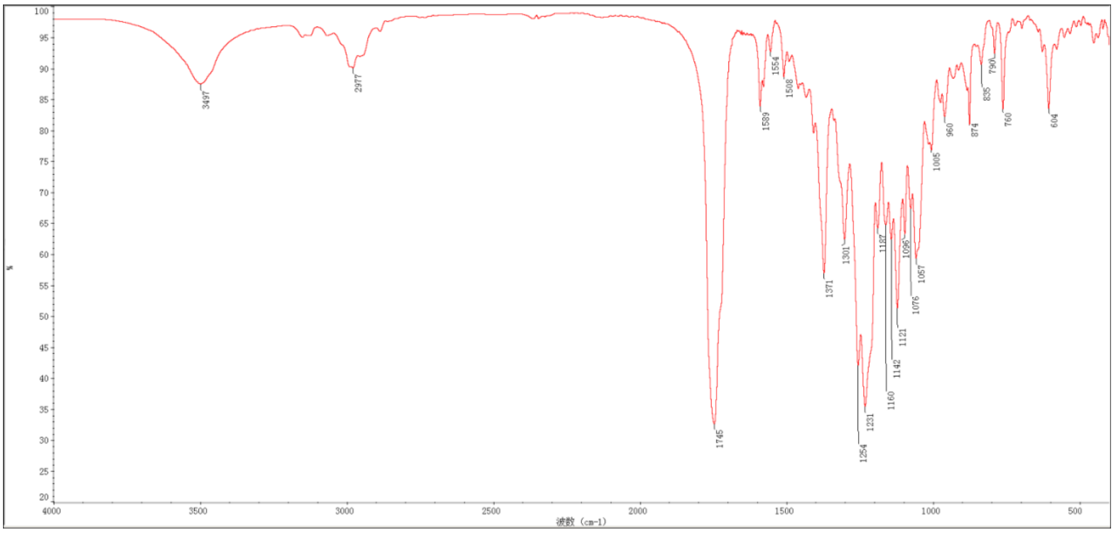

Figure S11. IR spectrum of compound **3** (KBr disc)

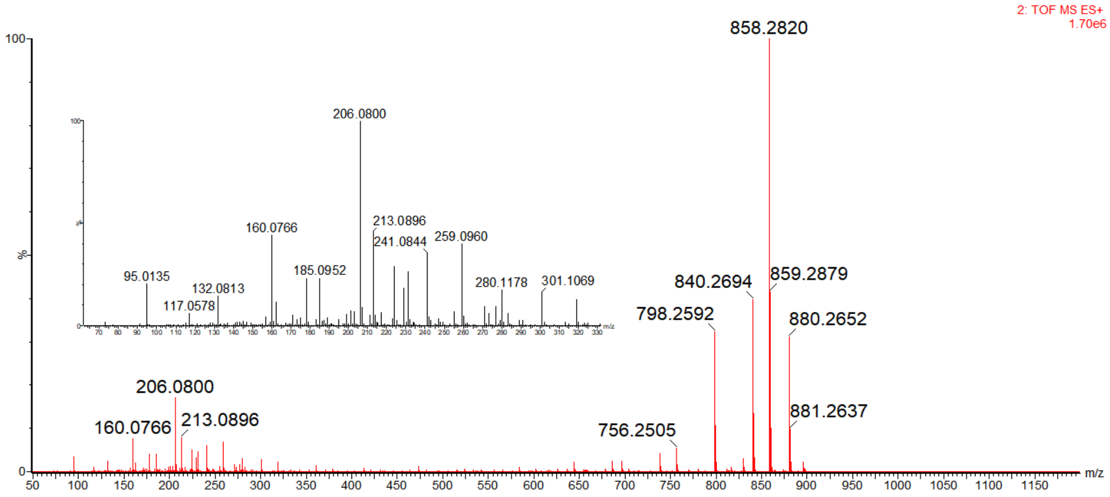

Figure S12. HRMS spectrum of compound **3**

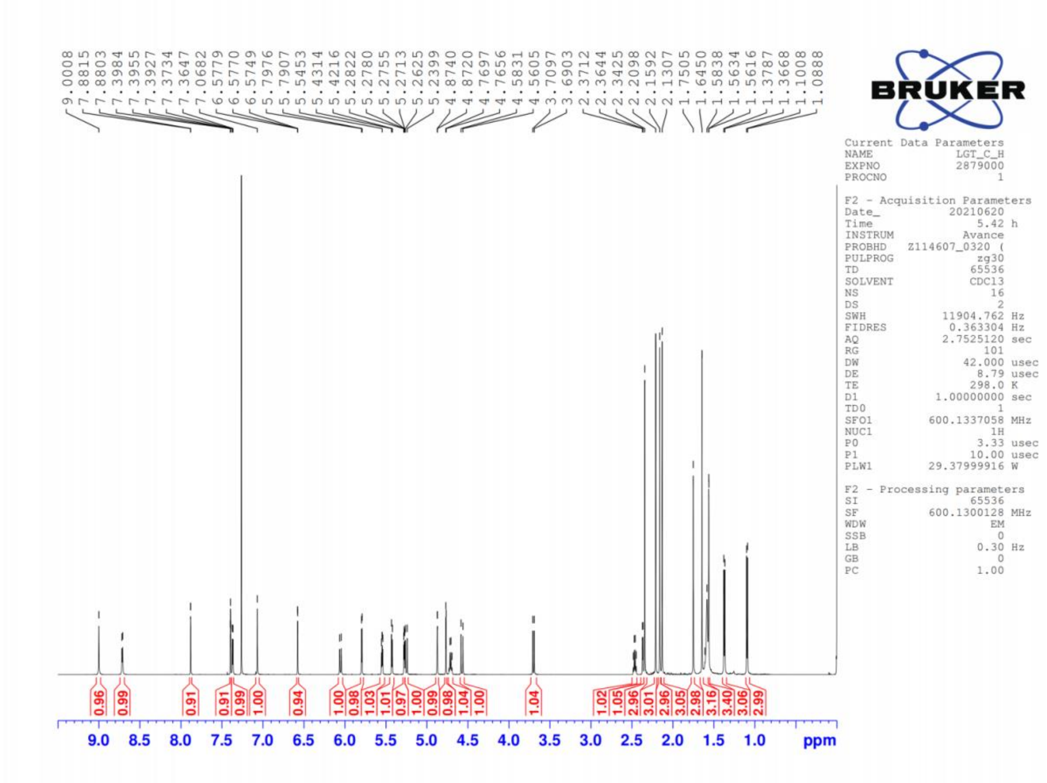

Figure S13. ^{1}H-NMR spectrum of compound **3**

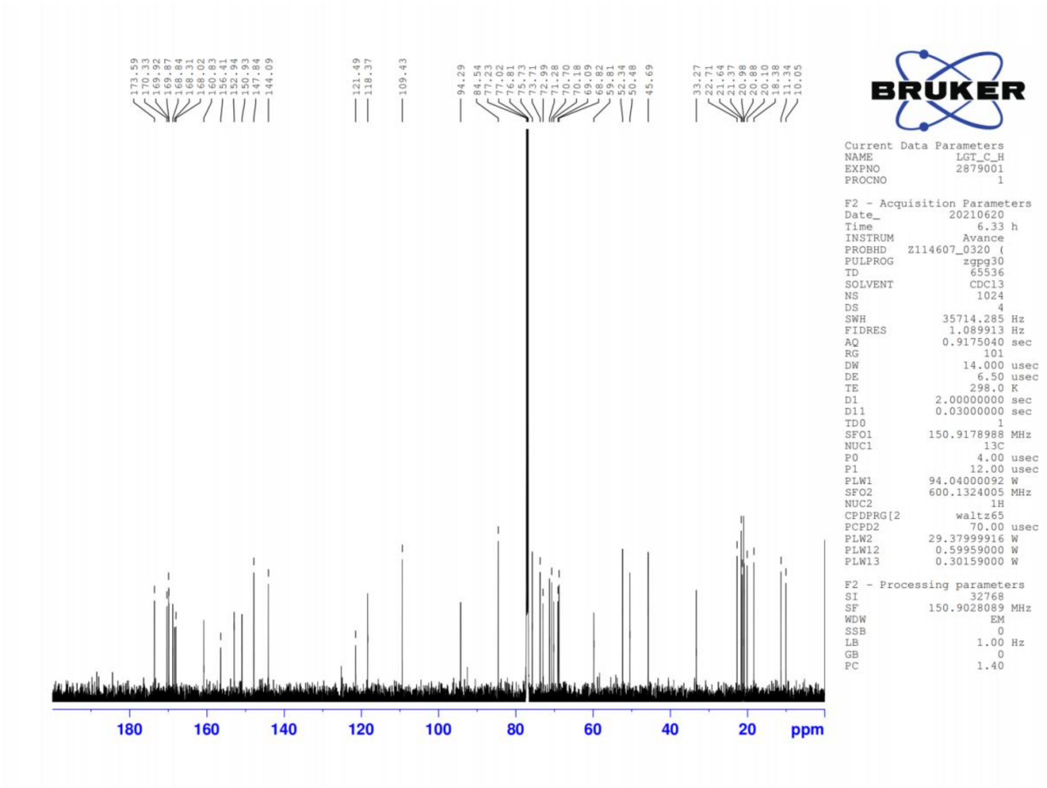

Figure S14. ^{13}C-NMR spectrum of compound **3**

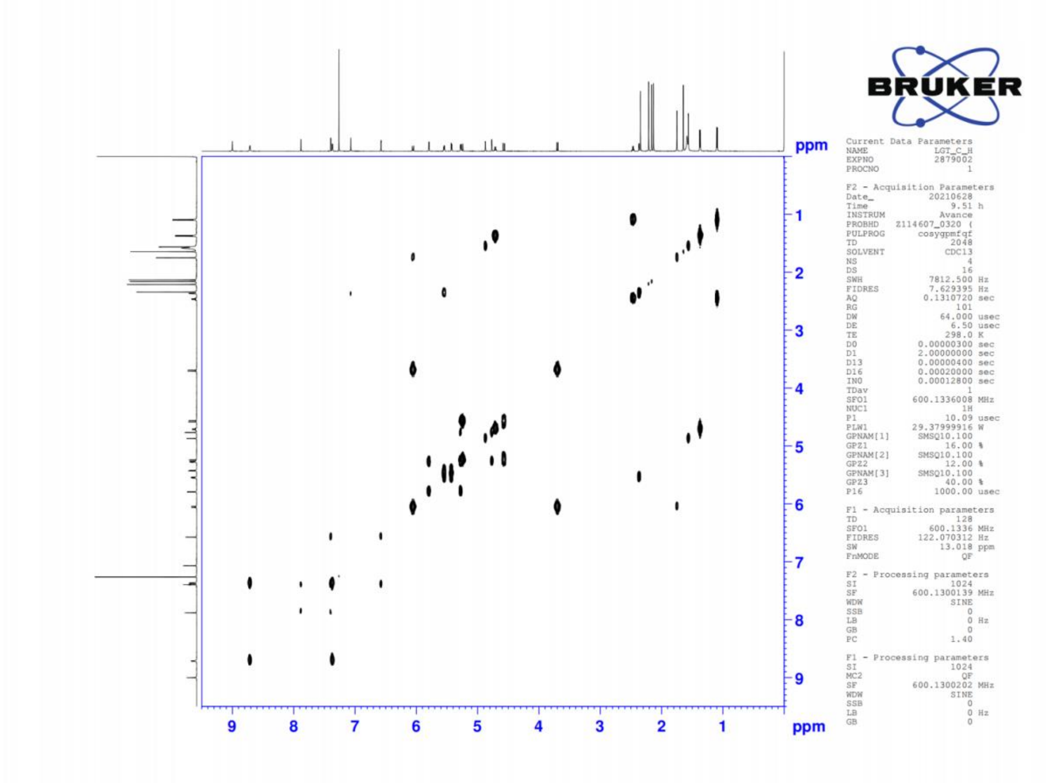

Figure S15. ^{1}H-^{1}H COSY spectrum of compound **3**

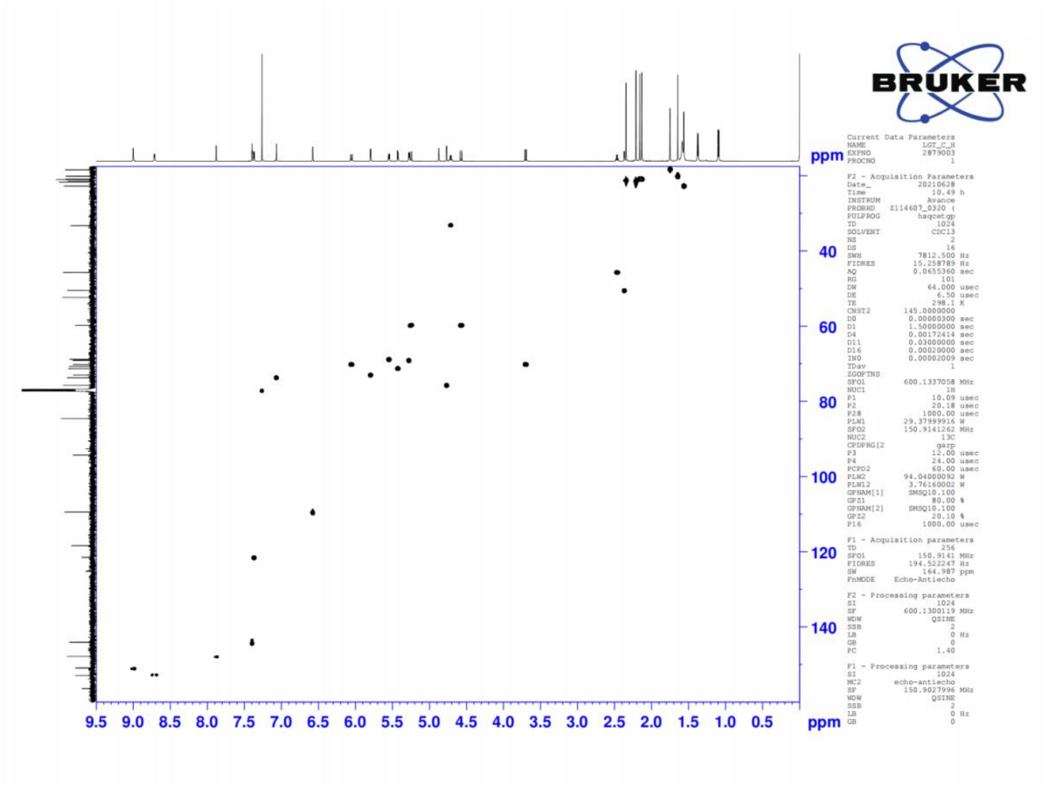

Figure S16. HSQC spectrum of compound **3**

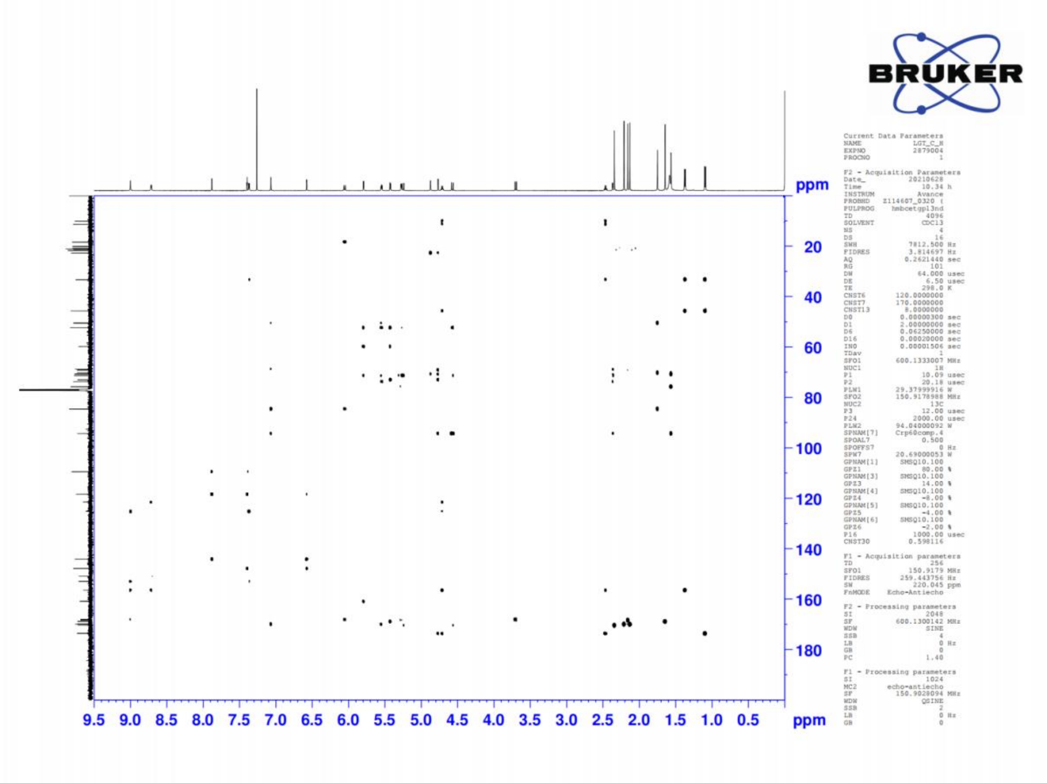

Figure S17. HMBC spectrum of compound **3**

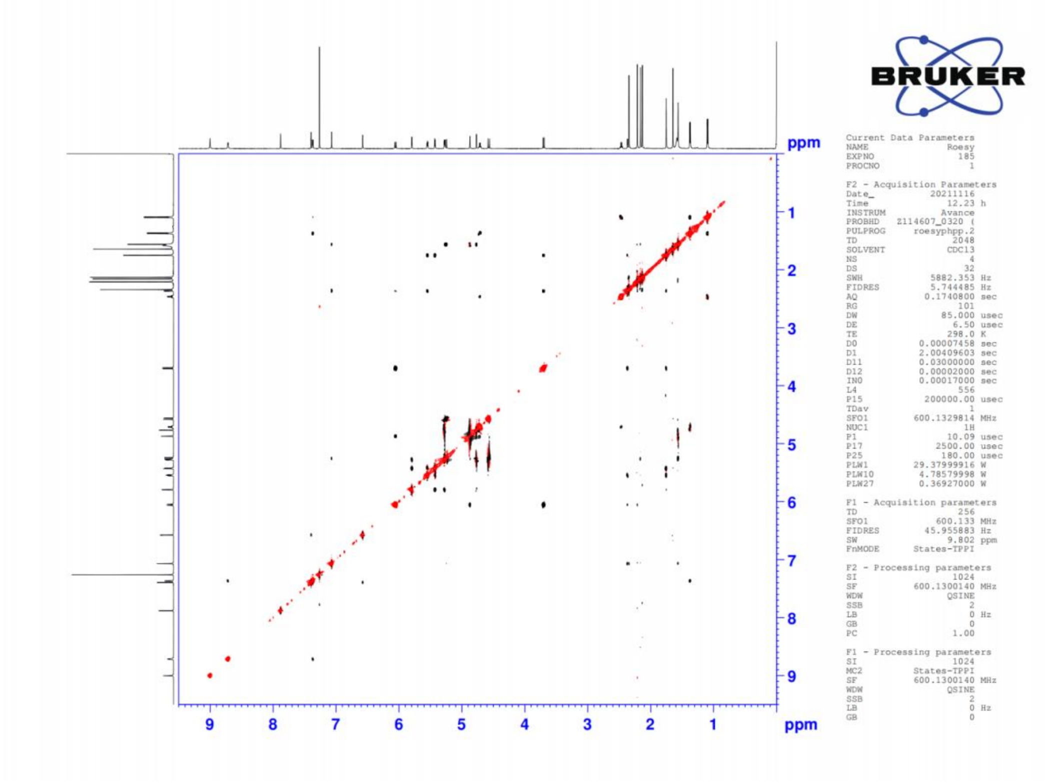

Figure S18. ROESY spectrum of compound **3**

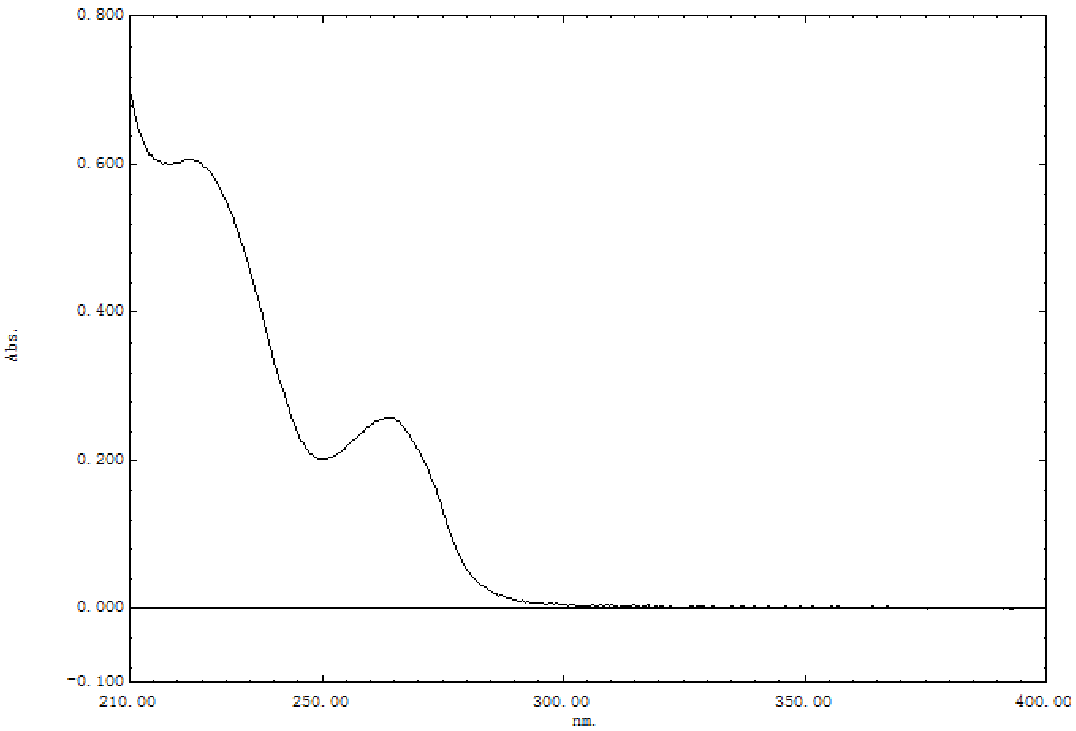

Figure S19. UV spectrum of compound **4** in methanol

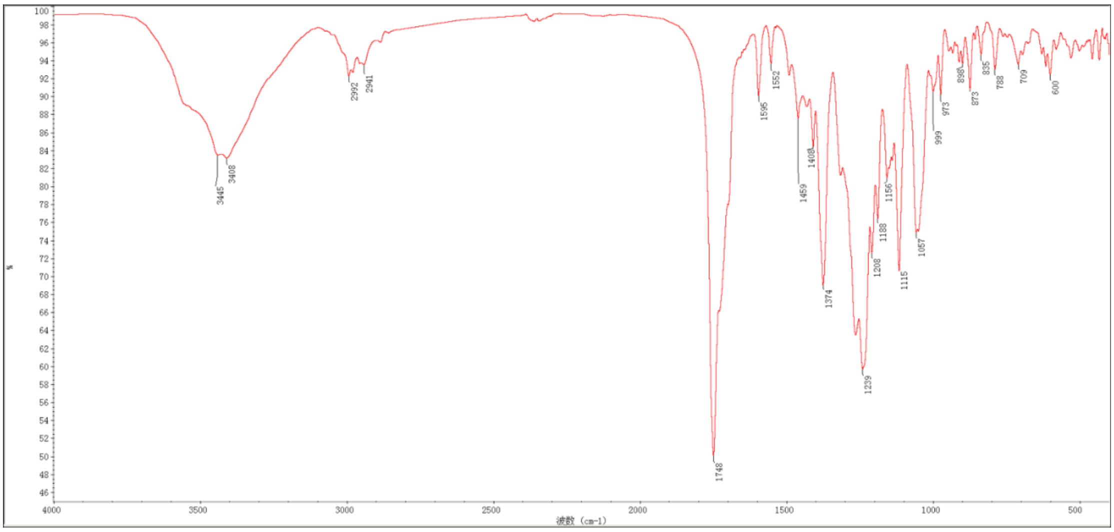

Figure S20. IR spectrum of compound **4** (KBr disc)

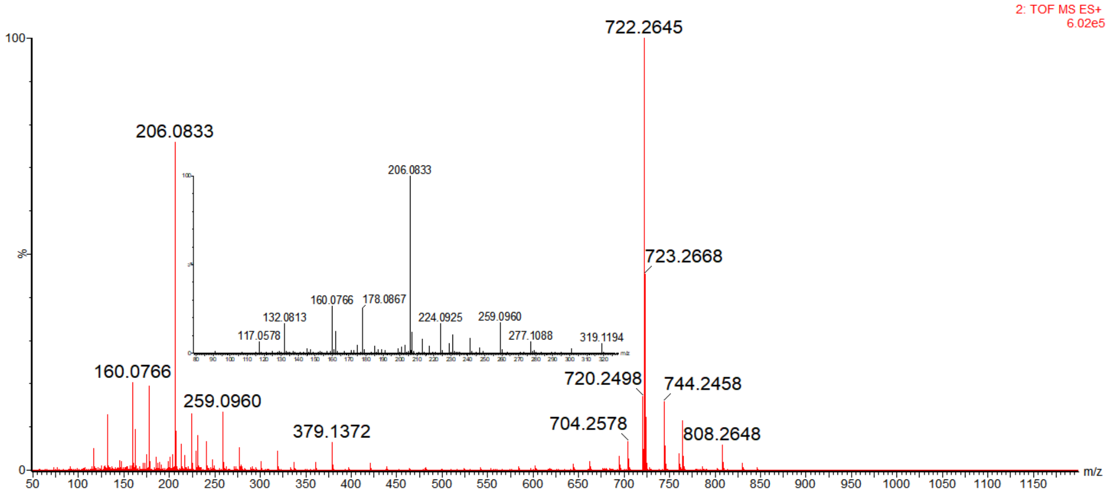

Figure S21. HRMS spectrum of compound **4**

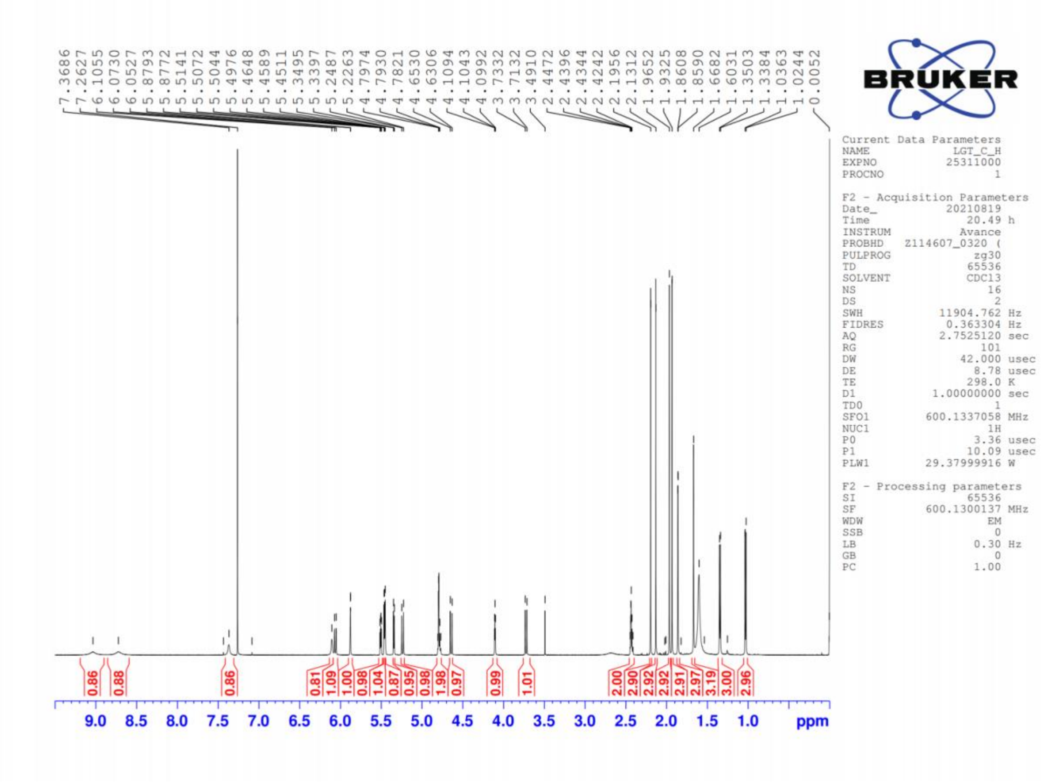

Figure S22. ^{1}H-NMR spectrum of compound **4**

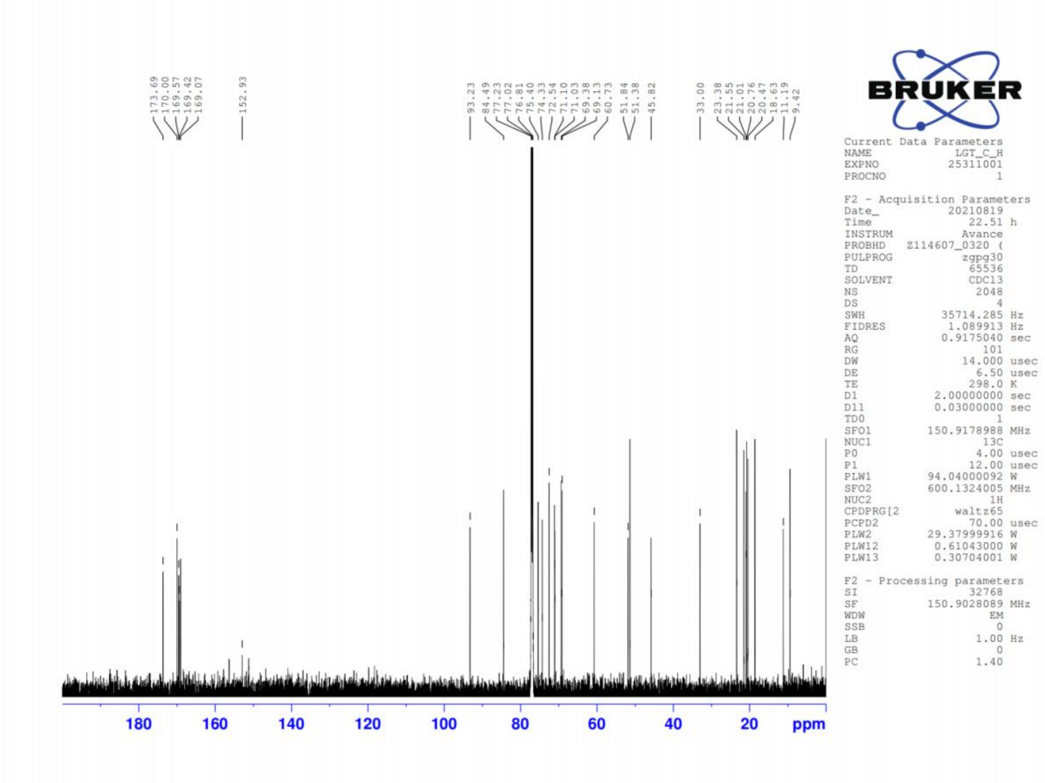

Figure S23. ^{13}C-NMR spectrum of compound **4**

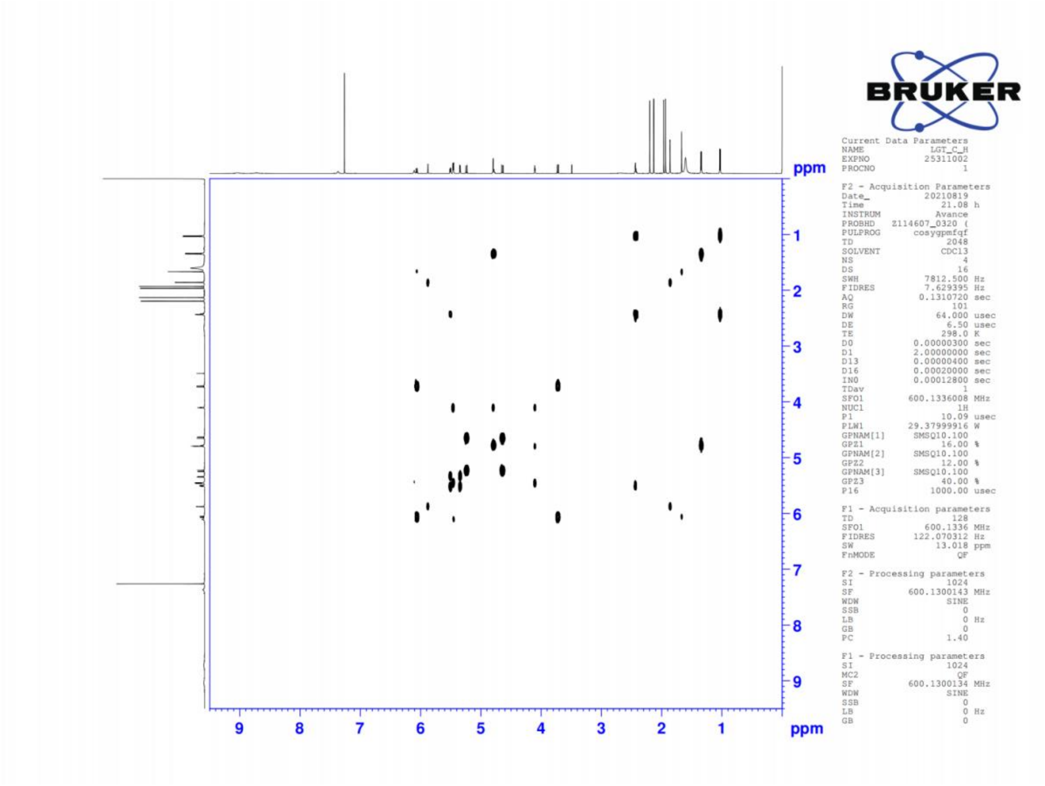

Figure S24. ^{1}H-^{1}H COSY spectrum of compound **4**

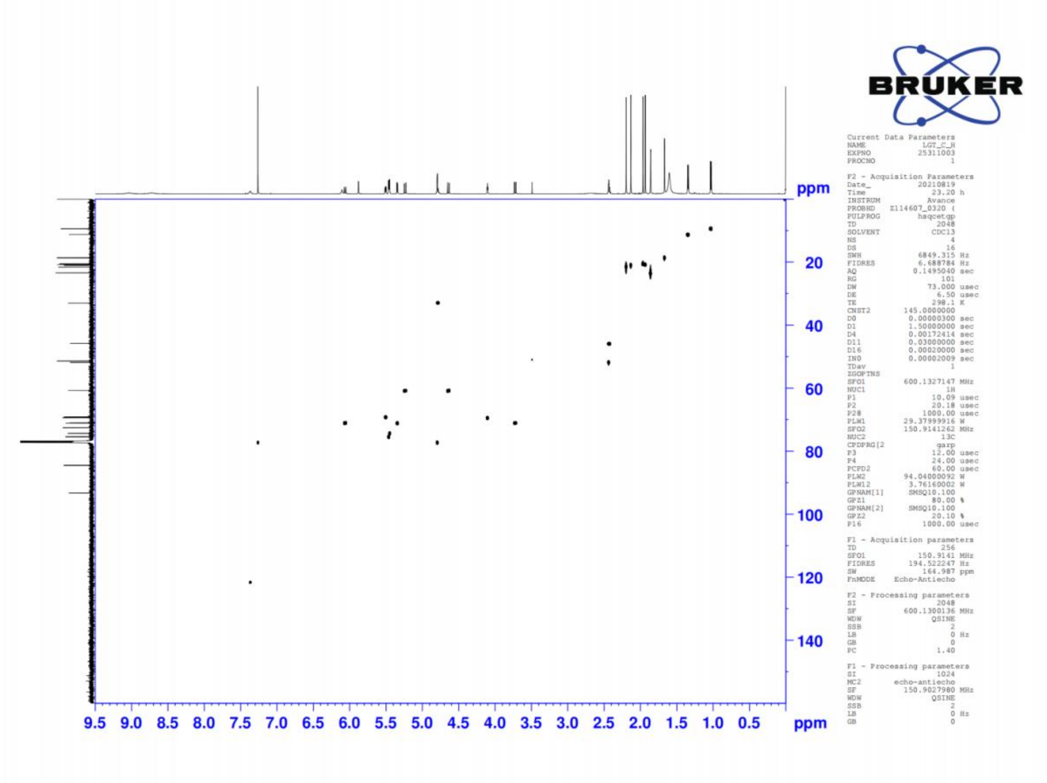

Figure S25. HSQC spectrum of compound **4**

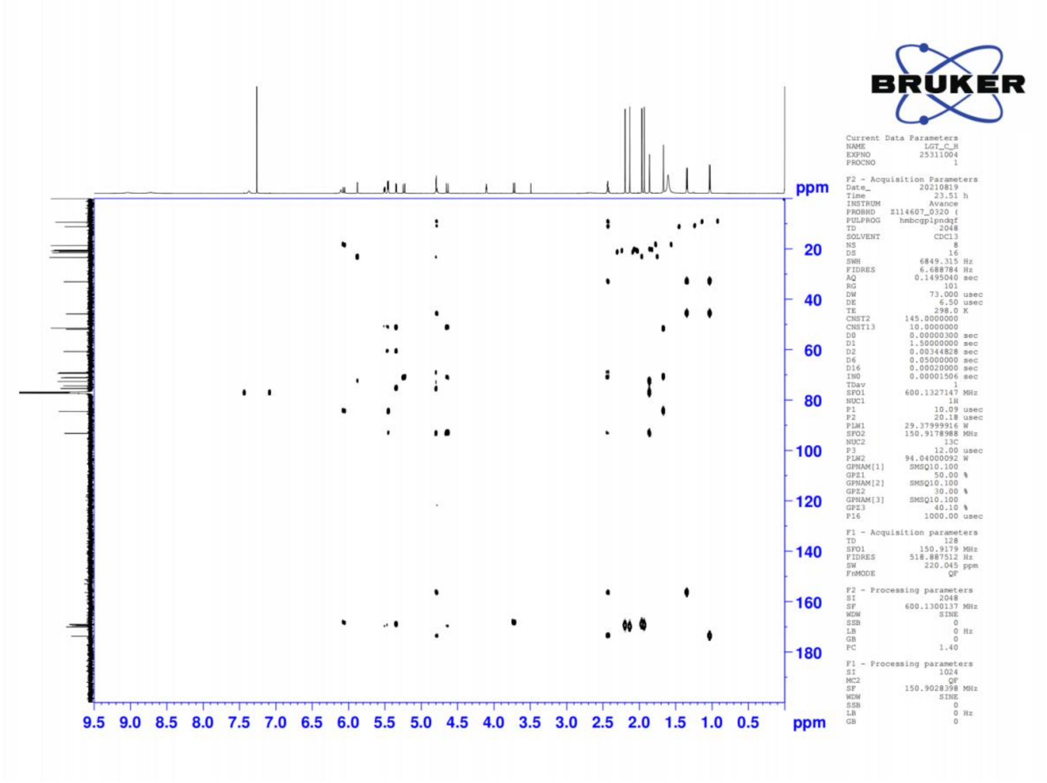

Figure S26. HMBC spectrum of compound **4**

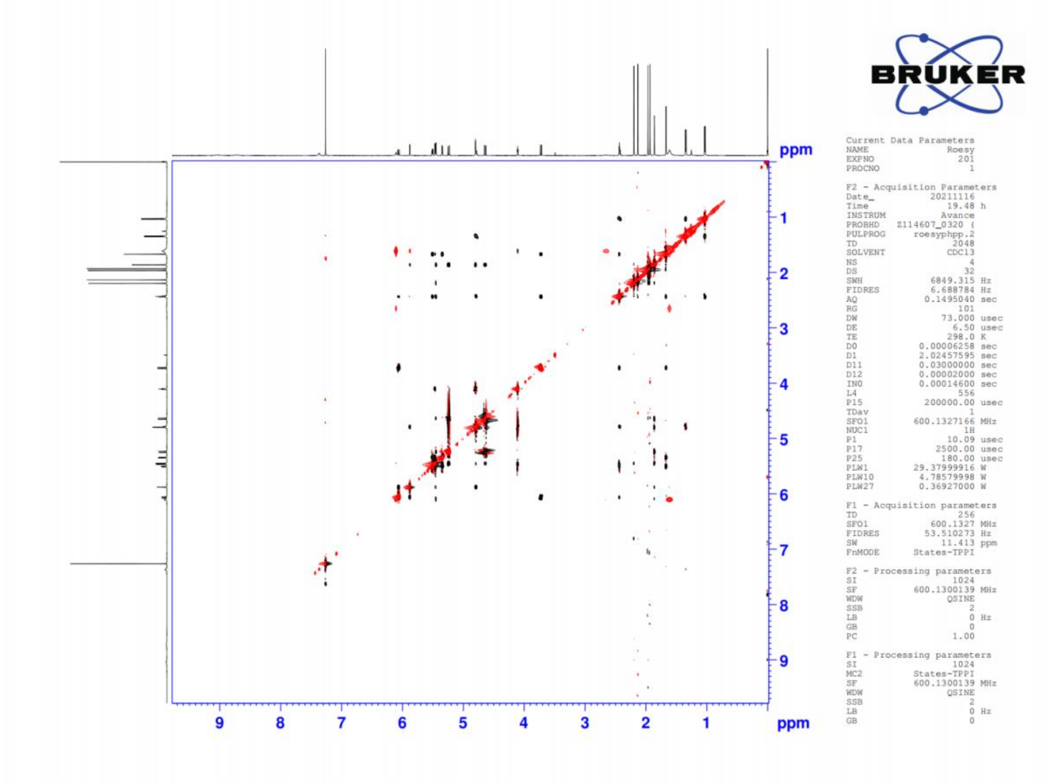

Figure S27. ROESY spectrum of compound **4**

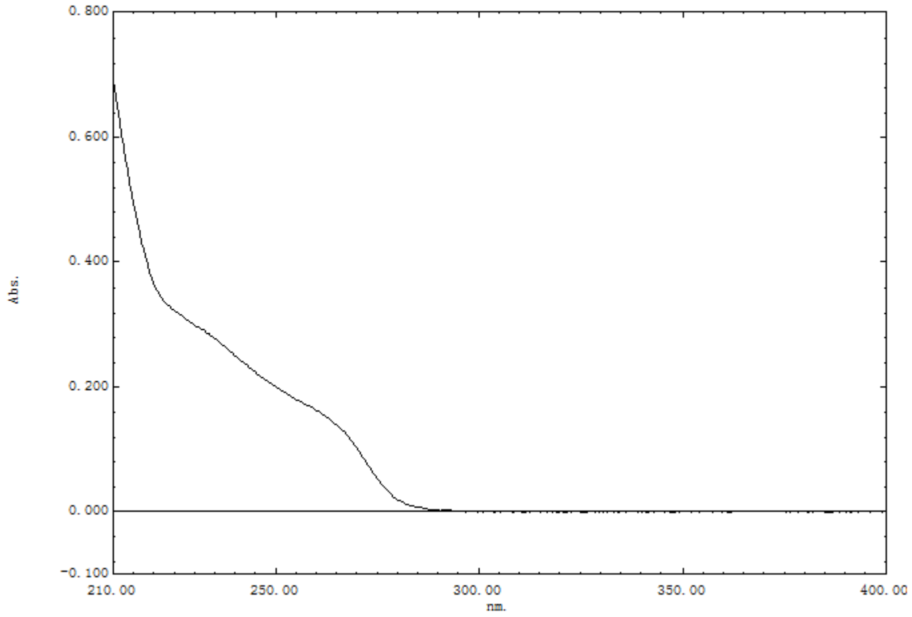

Figure S28. UV spectrum of compound **5** in methanol

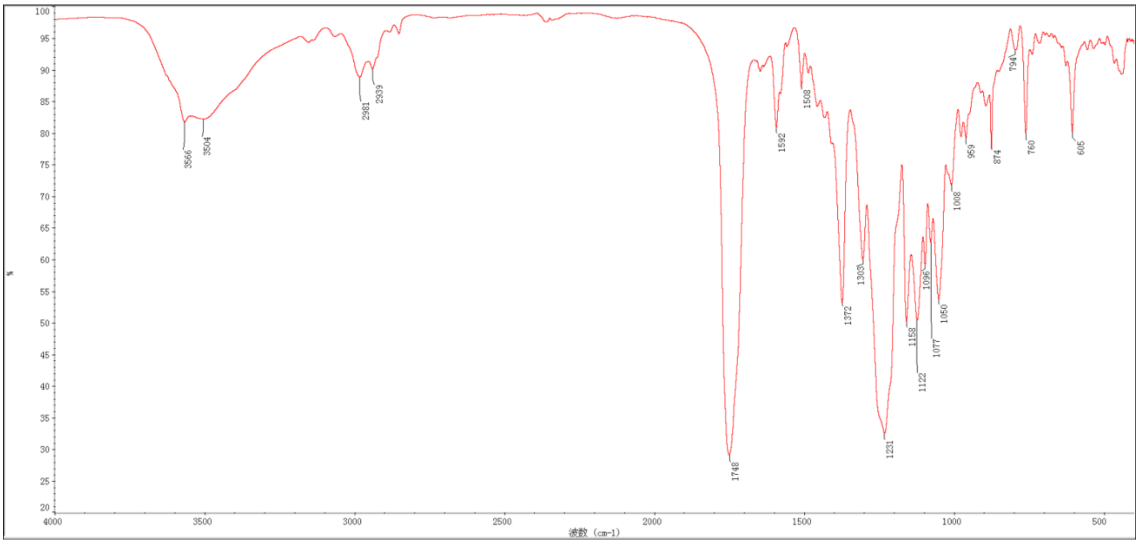

Figure S29. IR spectrum of compound **5** (KBr disc)

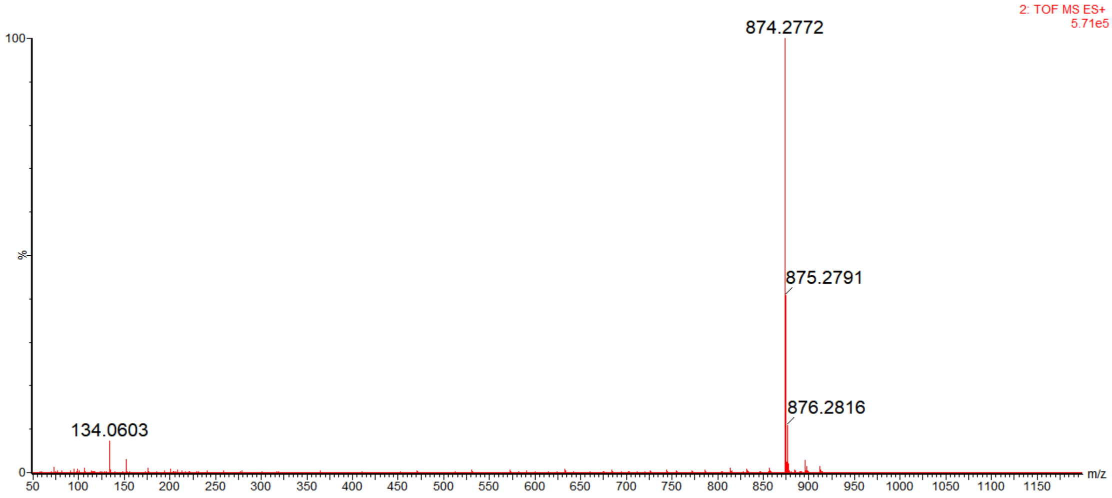

Figure S30. HRMS spectrum of compound **5**

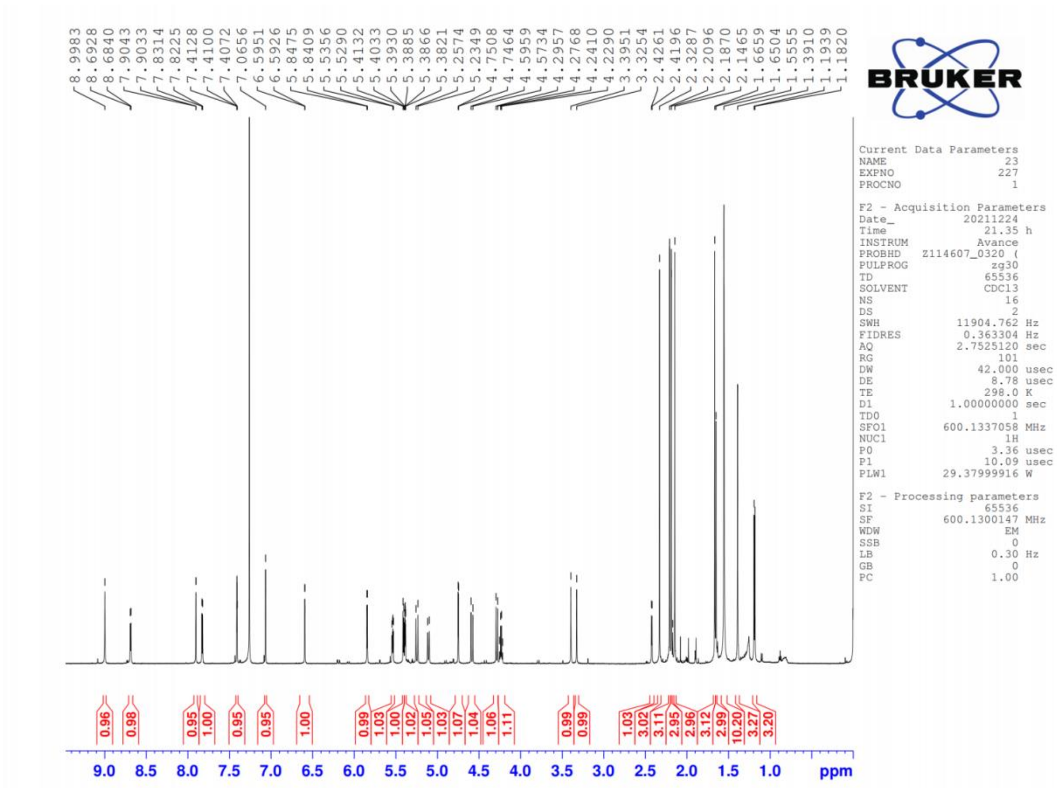

Figure S31. ^{1}H-NMR spectrum of compound **5**

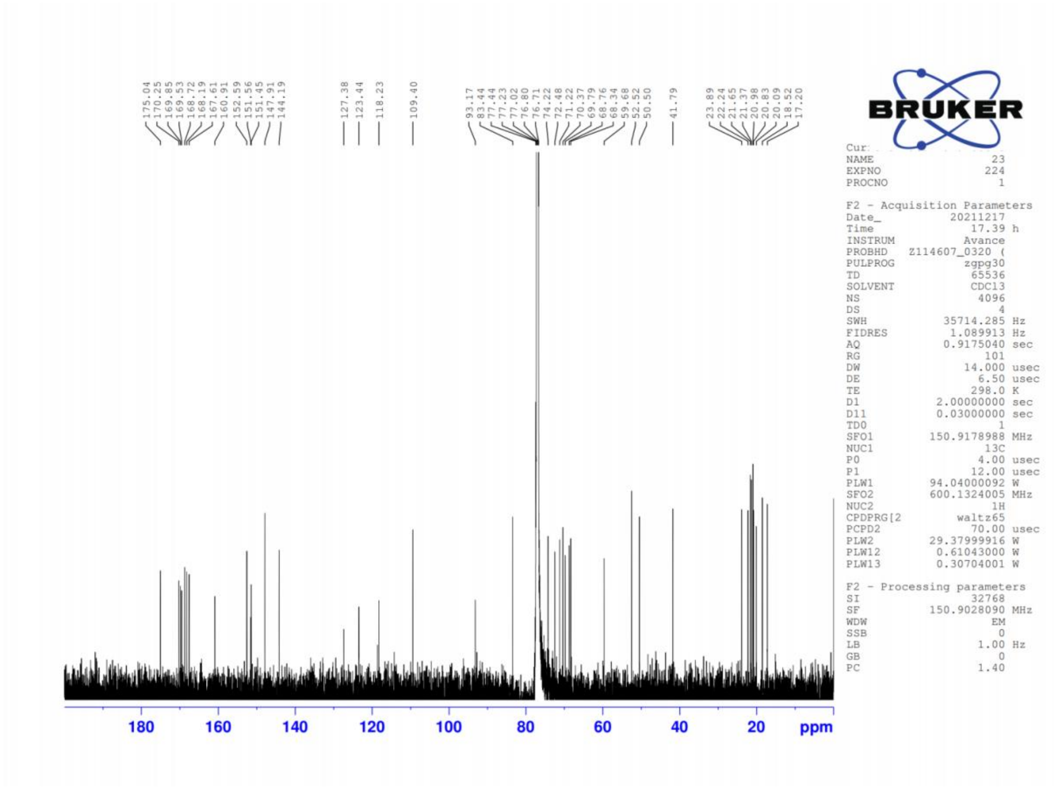

Figure S32. ^{13}C-NMR spectrum of compound **5**

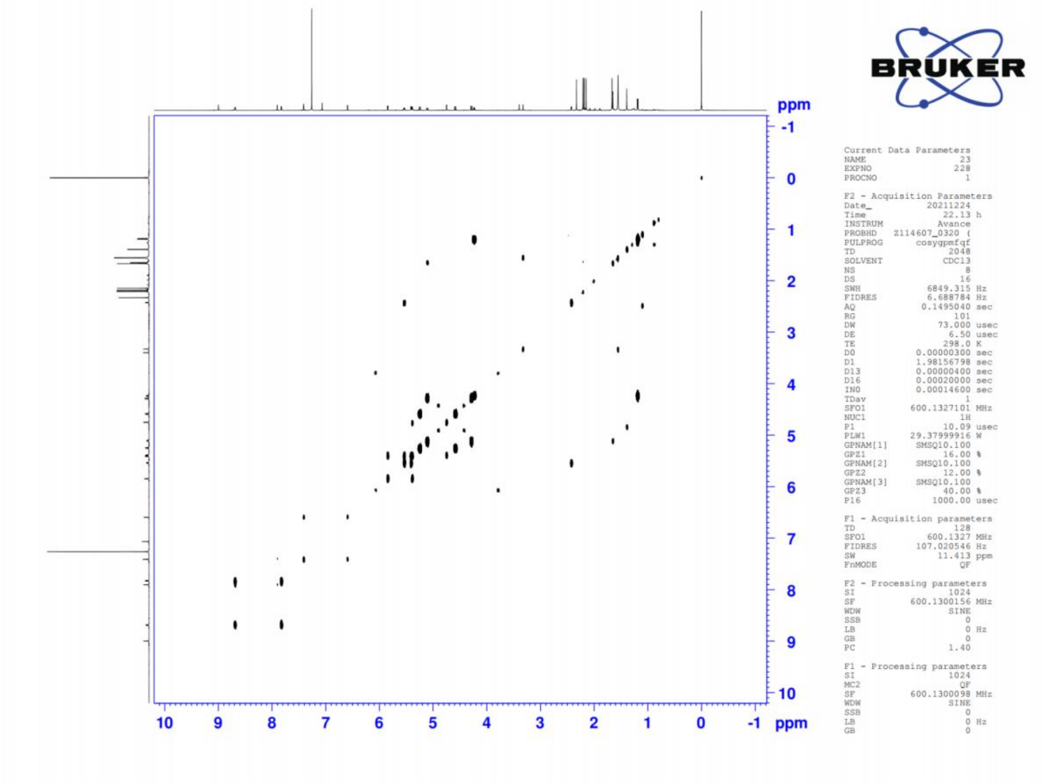

Figure S33. ^{1}H-^{1}H COSY spectrum of compound **5**

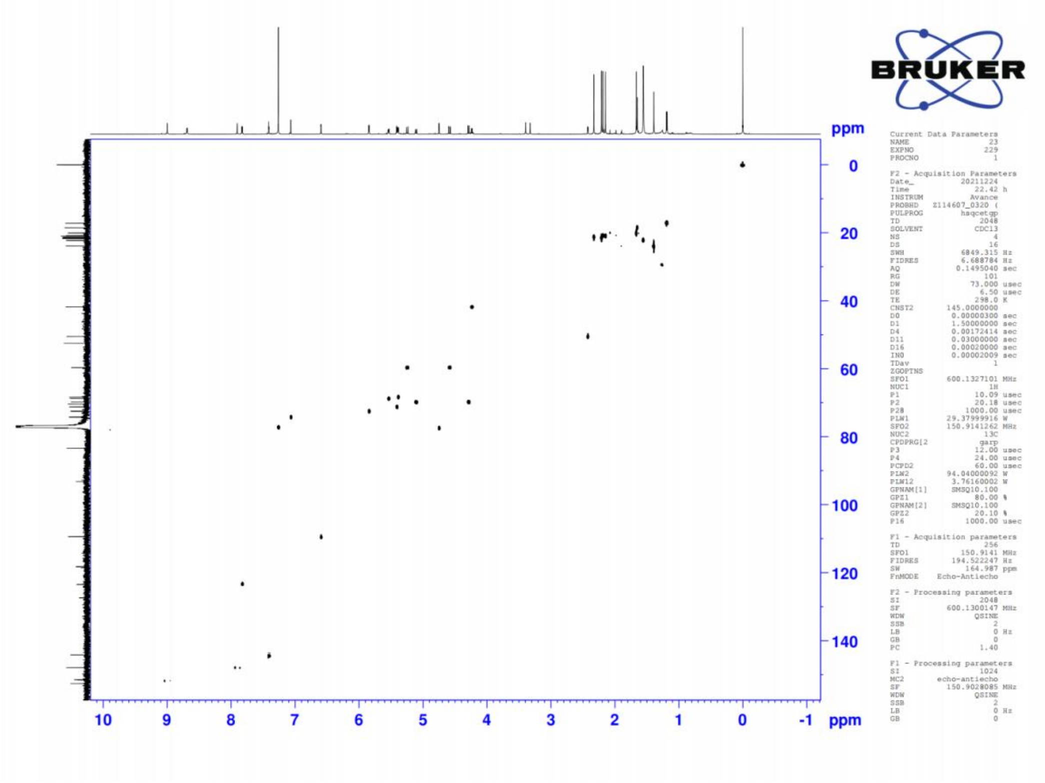

Figure S34. HSQC spectrum of compound **5**

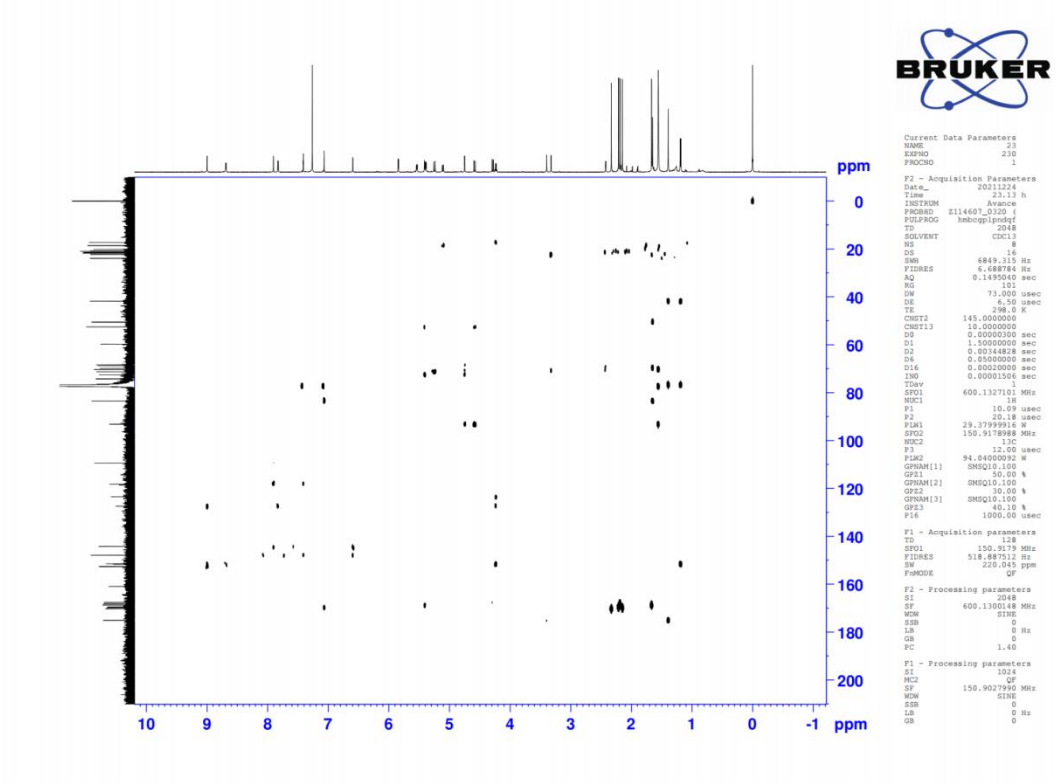

Figure S35. HMBC spectrum of compound **5**

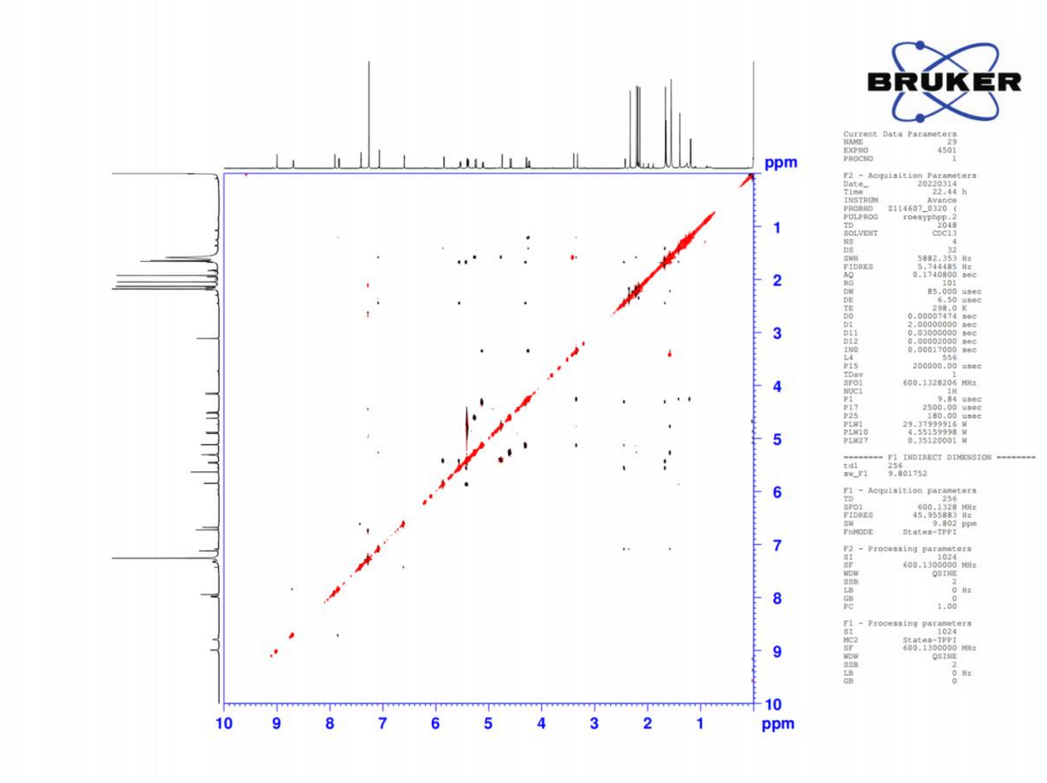

Figure S36. ROESY spectrum of compound **5**

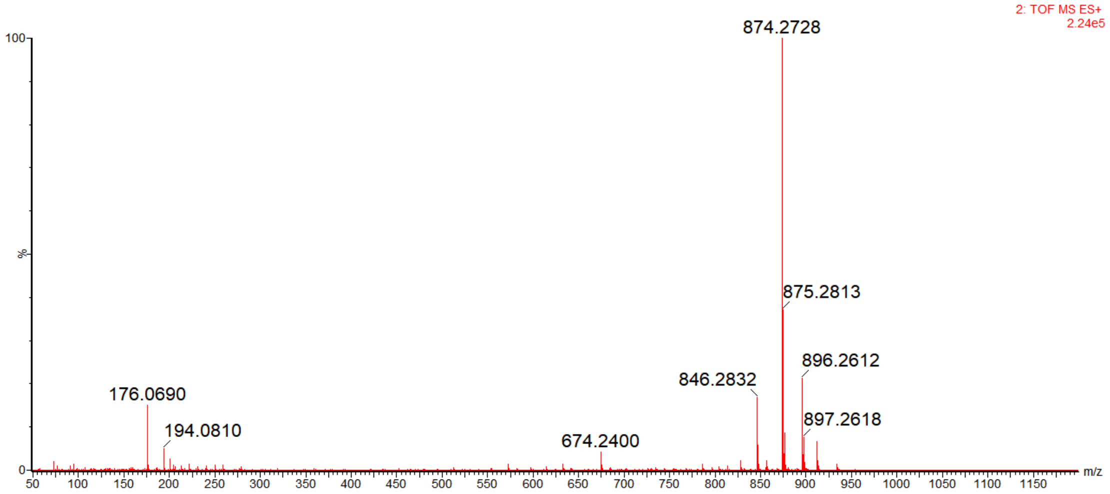

Figure S37. HRMS spectrum of compound **2**

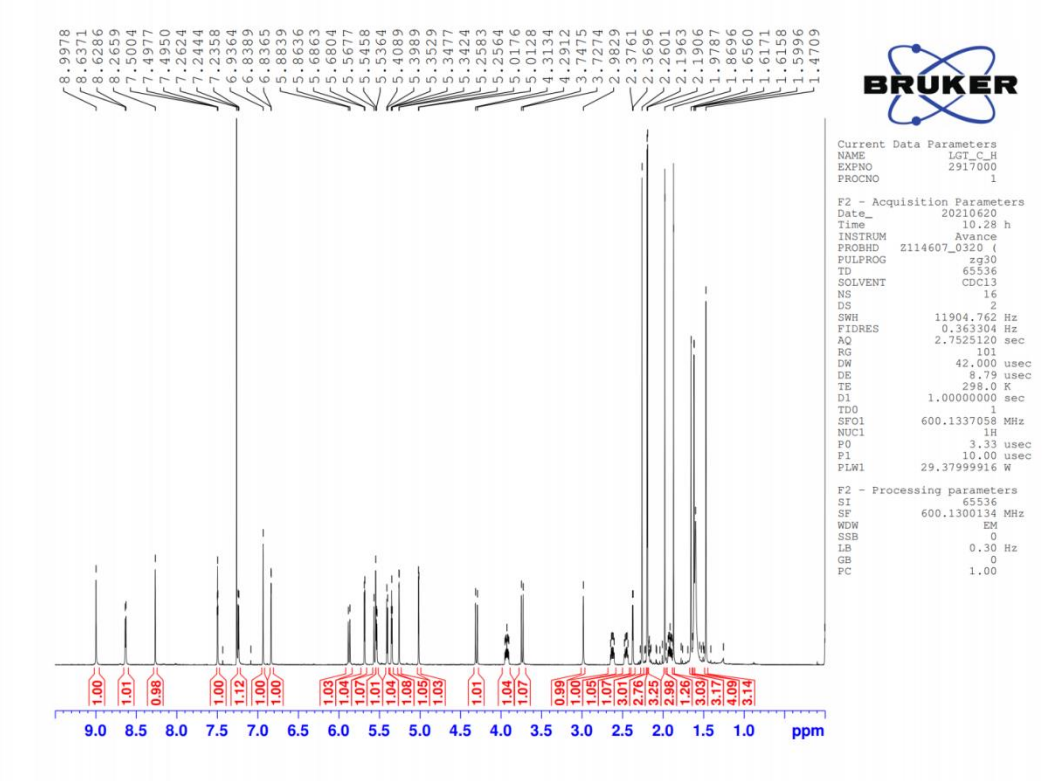

Figure S38. ^{1}H-NMR spectrum of compound **2**

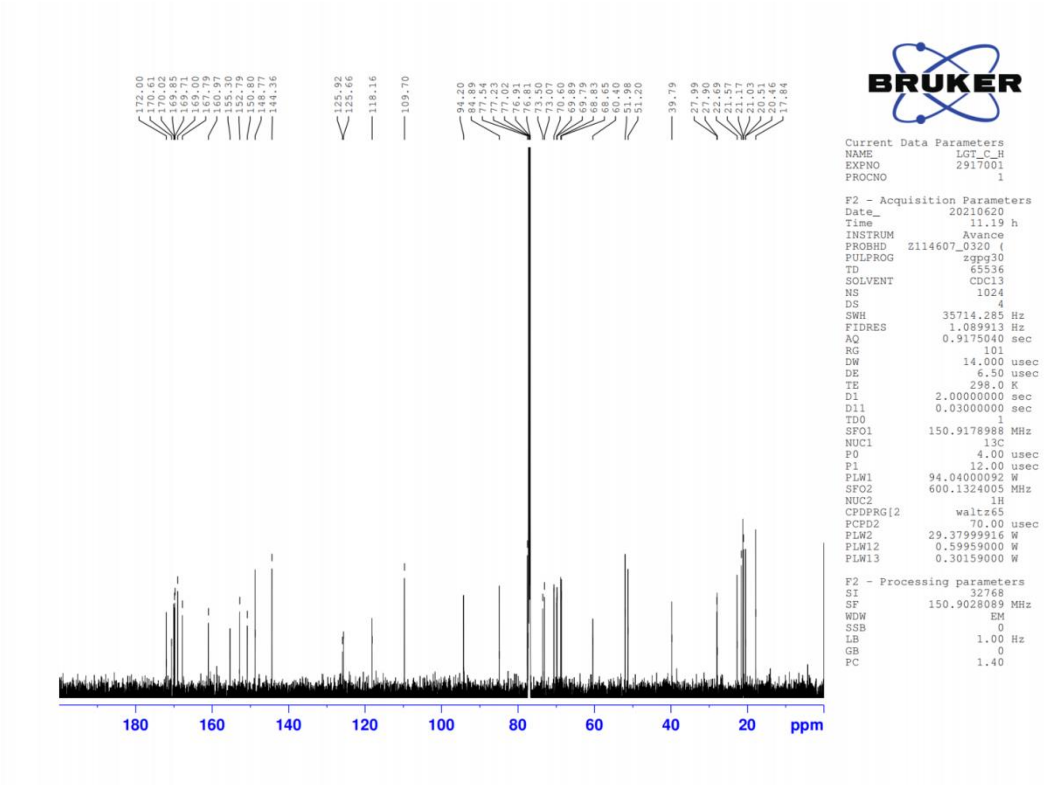

Figure S39. ^{13}C-NMR spectrum of compound **2**

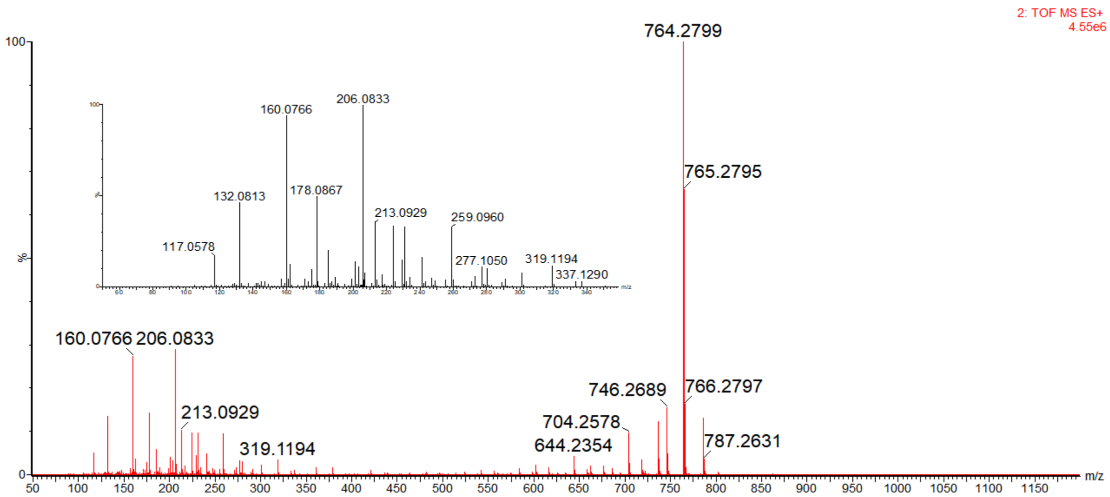

Figure S40. HRMS spectrum of compound **6**

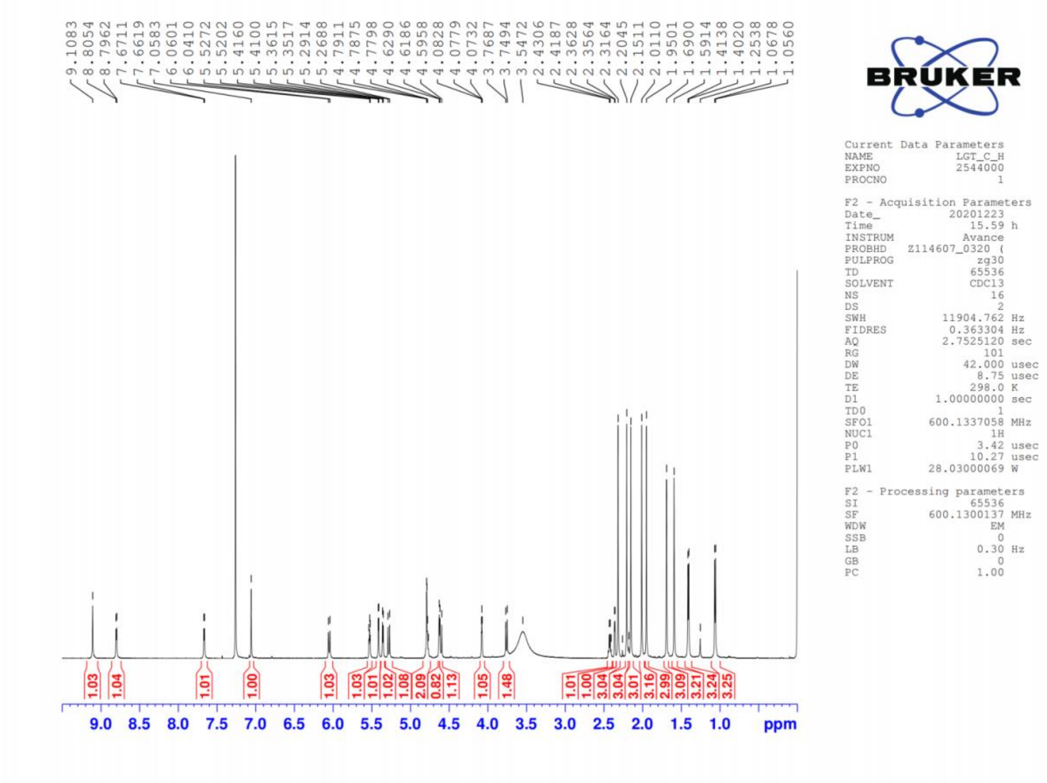

Figure S41. ^{1}H-NMR spectrum of compound **6**

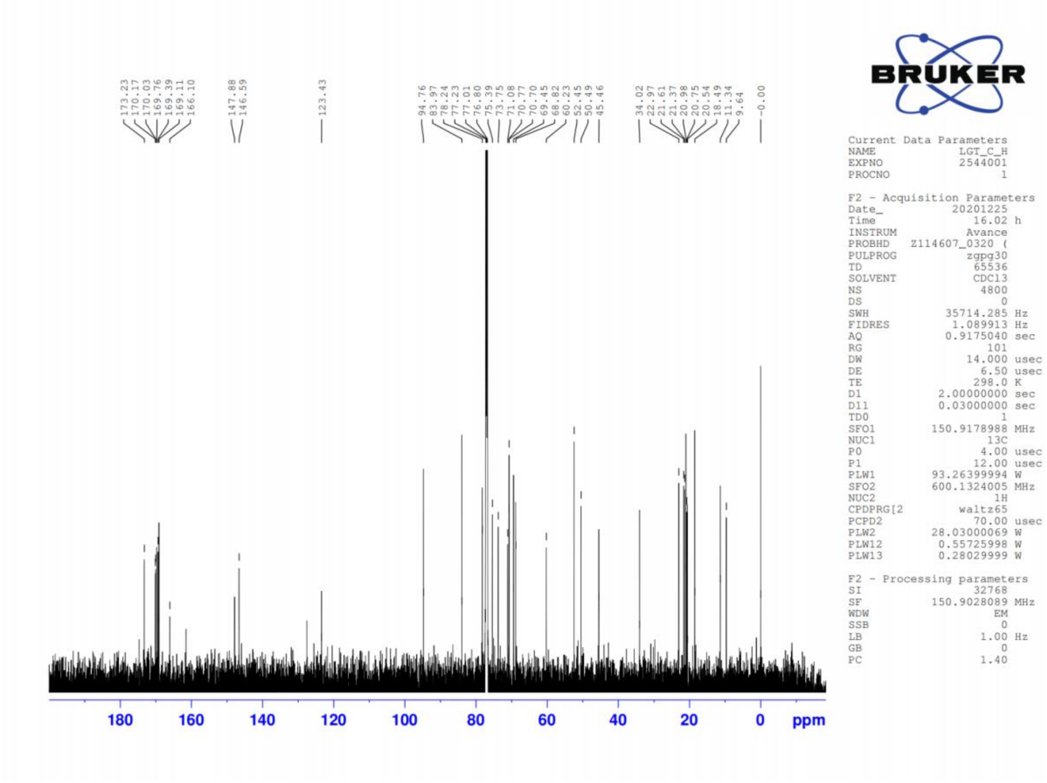

Figure S42. ^{13}C-NMR spectrum of compound **6**

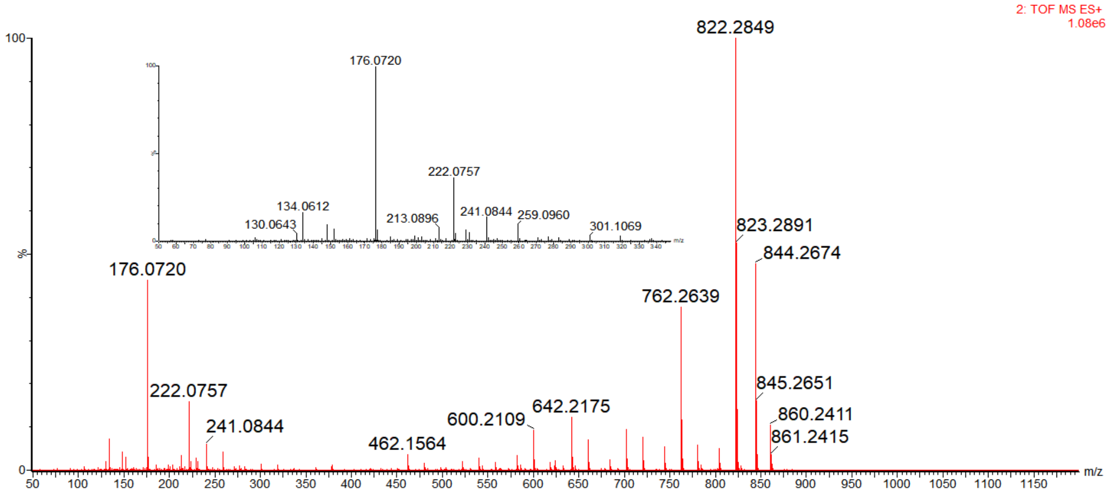

Figure S43. HRMS spectrum of compound **7**

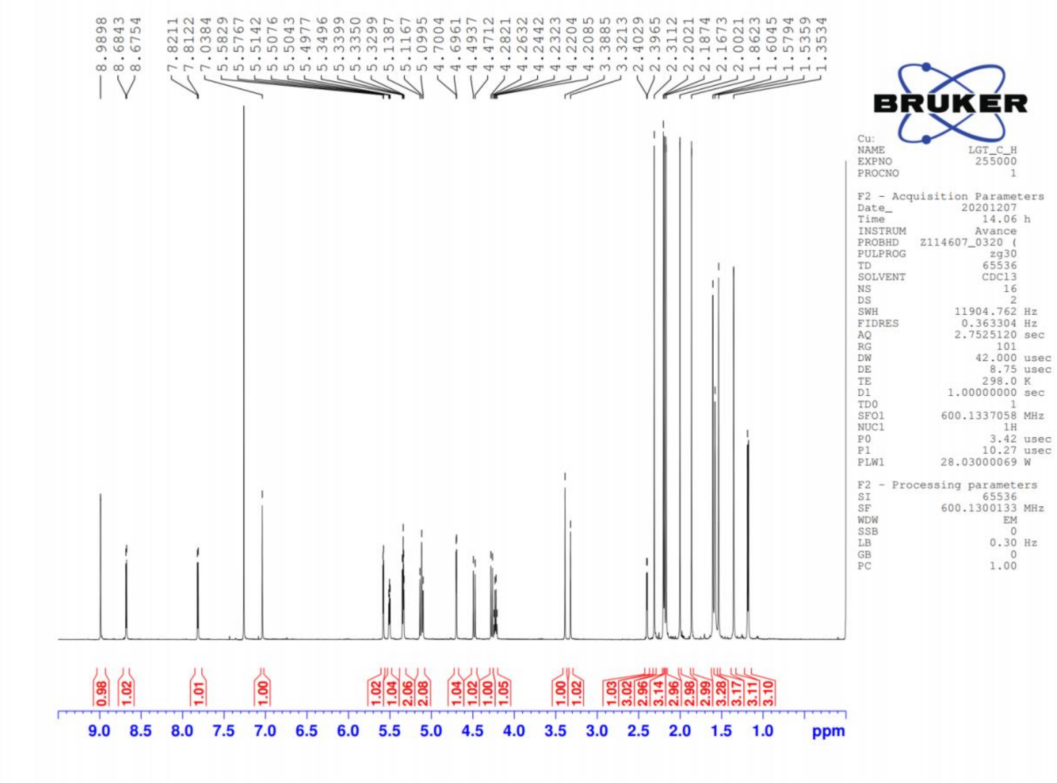

Figure S44. ^{1}H-NMR spectrum of compound **7**

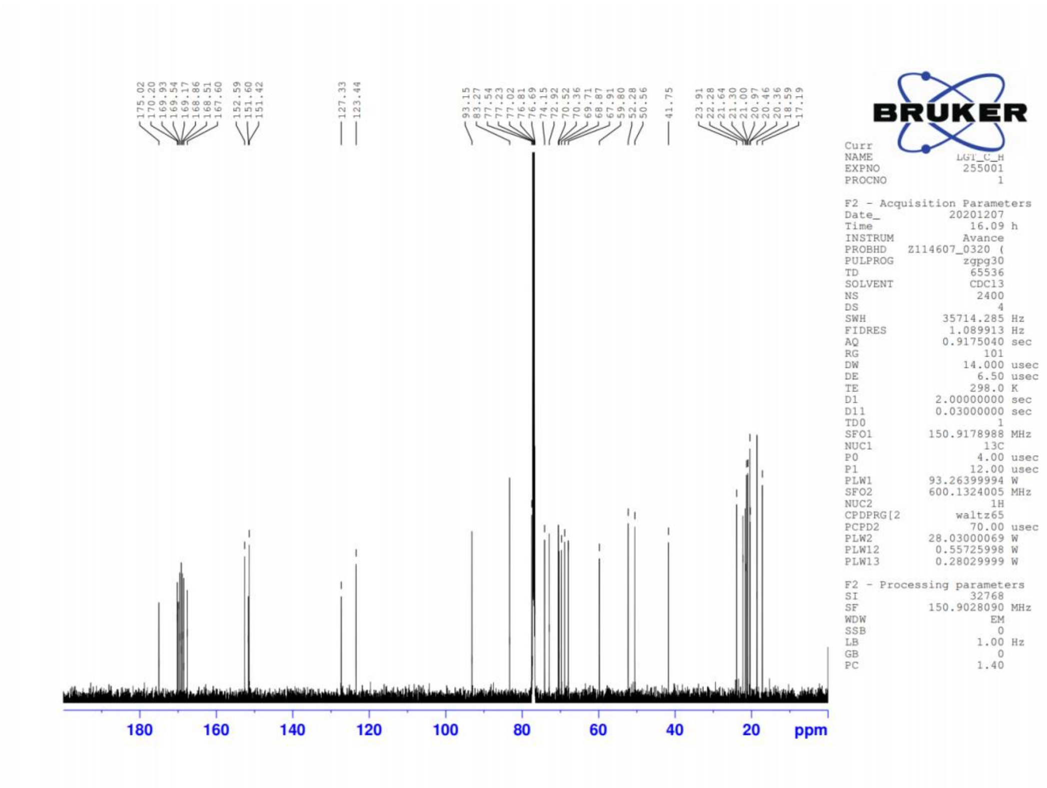

Figure S45. ^{13}C-NMR spectrum of compound **7**

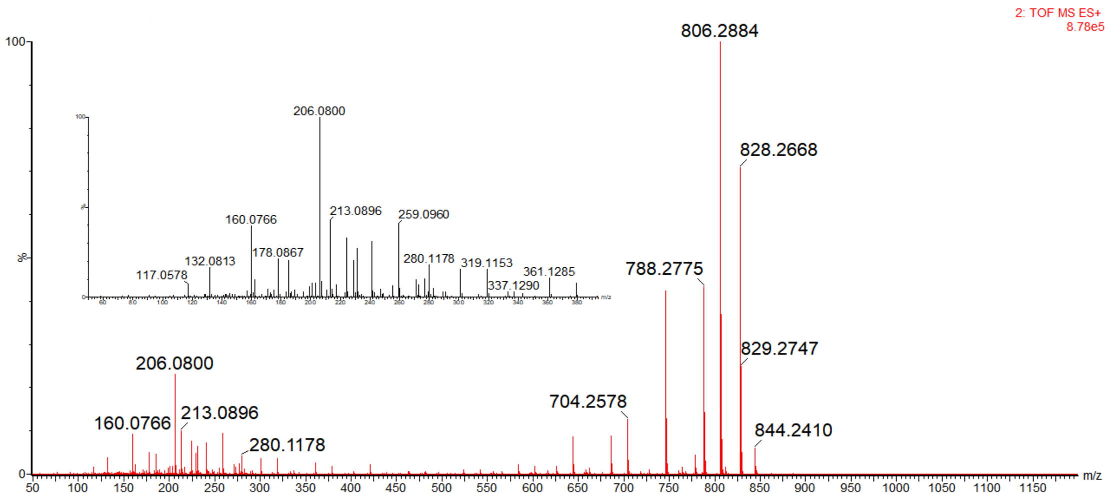

Figure S46. HRMS spectrum of compound **8**

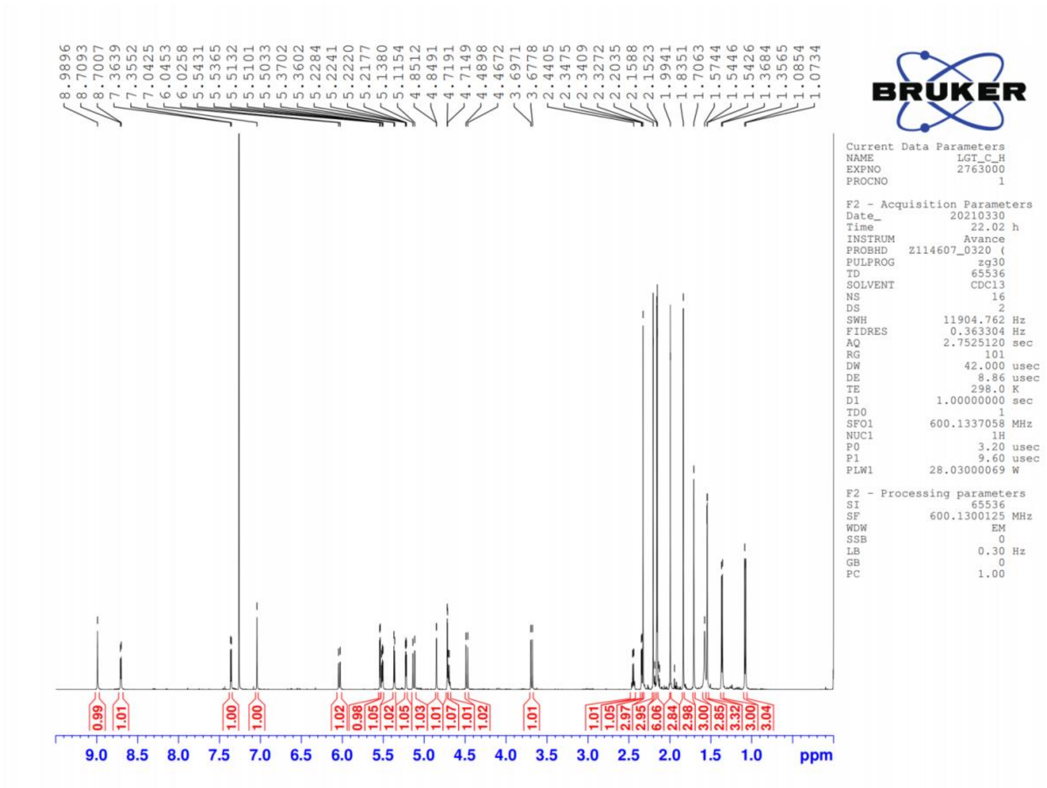

Figure S47. ^{1}H-NMR spectrum of compound **8**

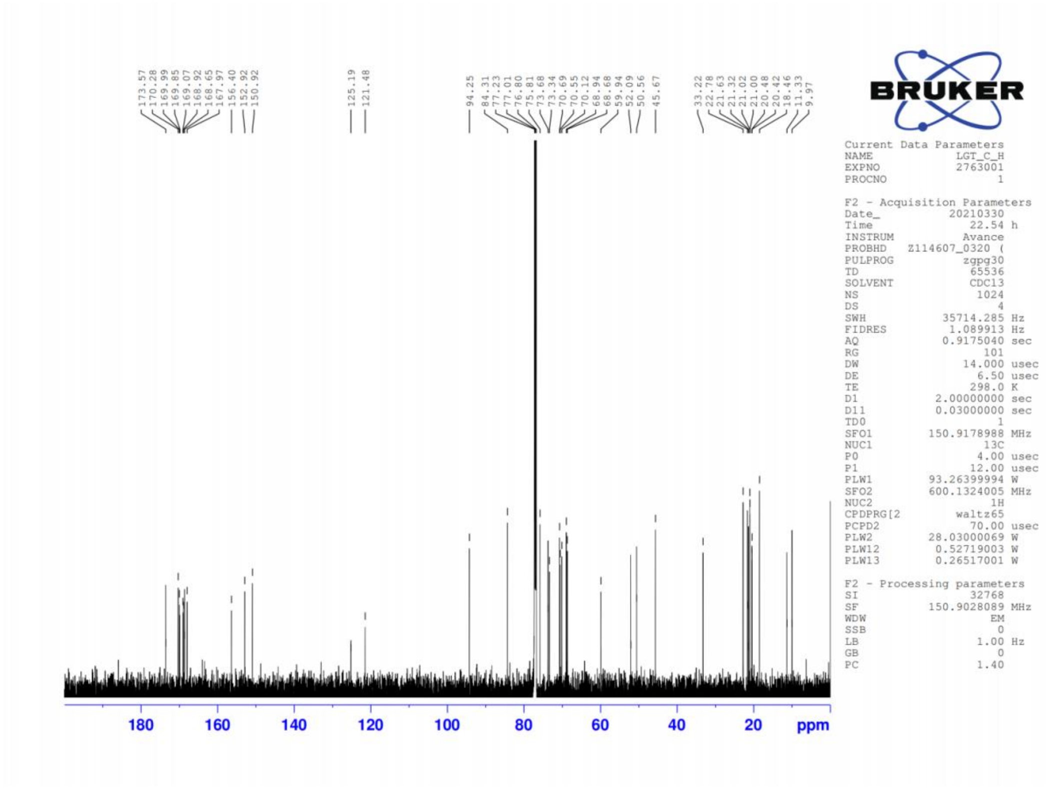

Figure S48. ^{13}C-NMR spectrum of compound **8**

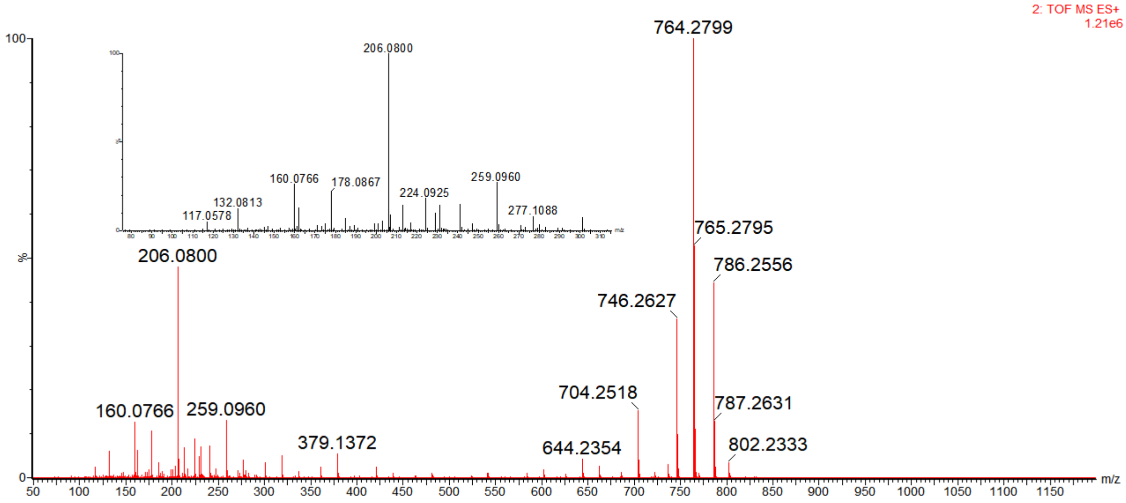

Figure S49. HRMS spectrum of compound **9**

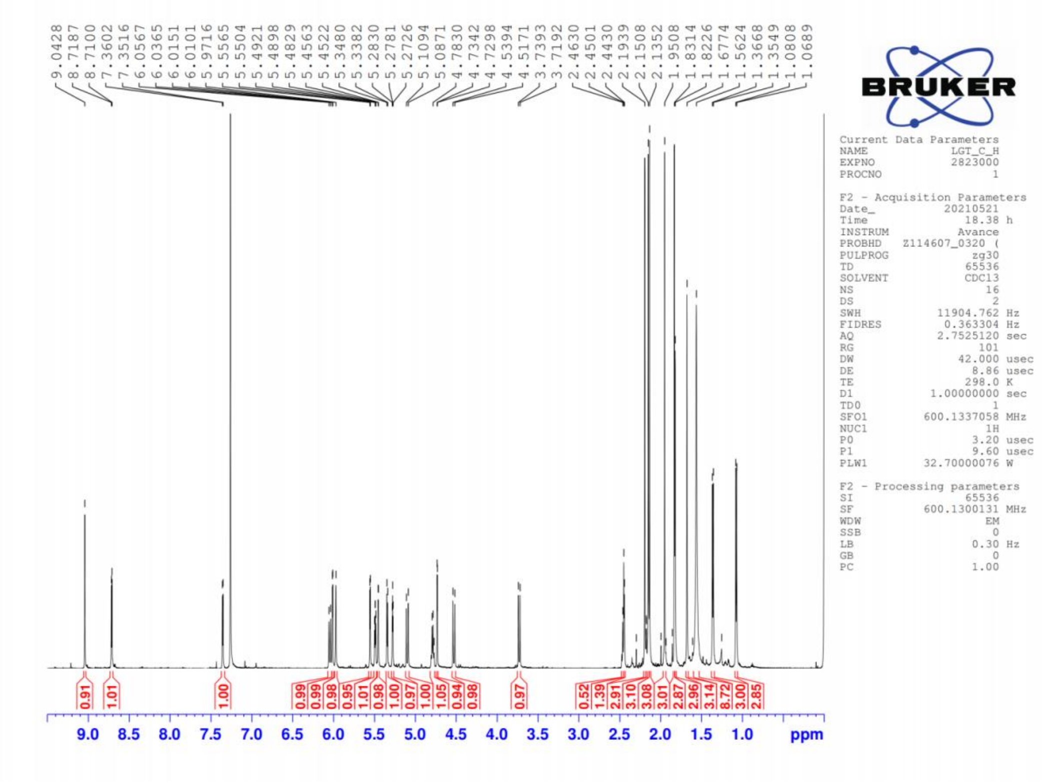

Figure S50. ^{1}H-NMR spectrum of compound **9**

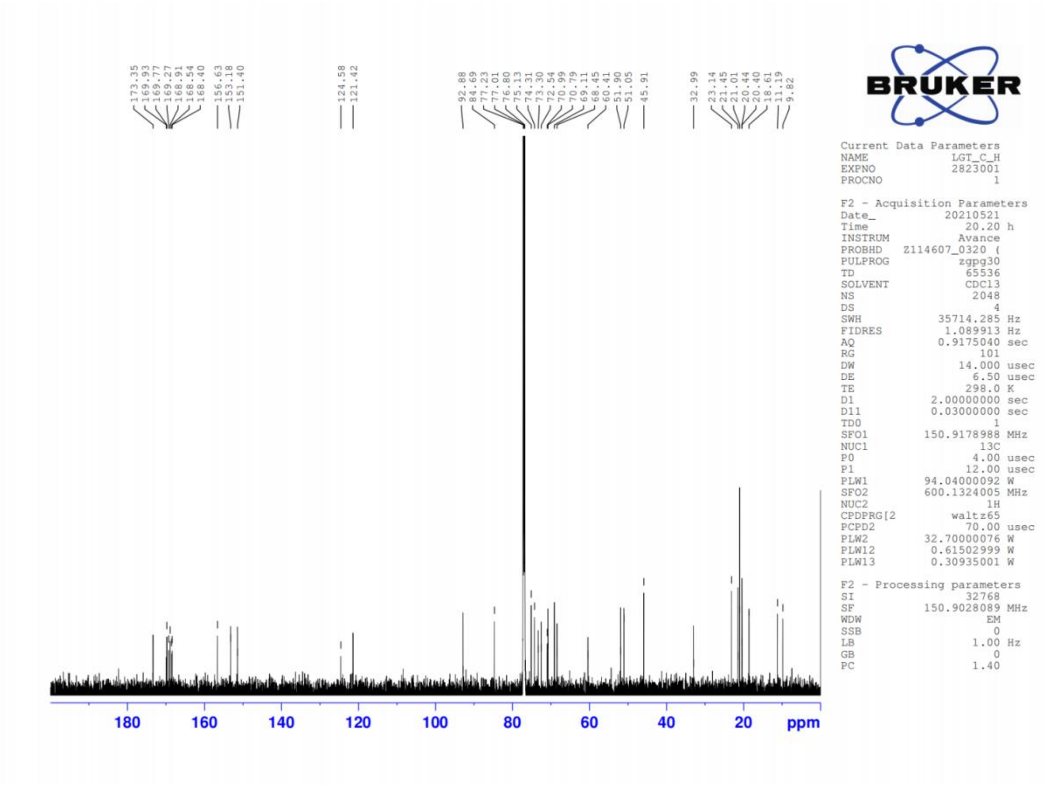

Figure S51. ^{13}C-NMR spectrum of compound **9**

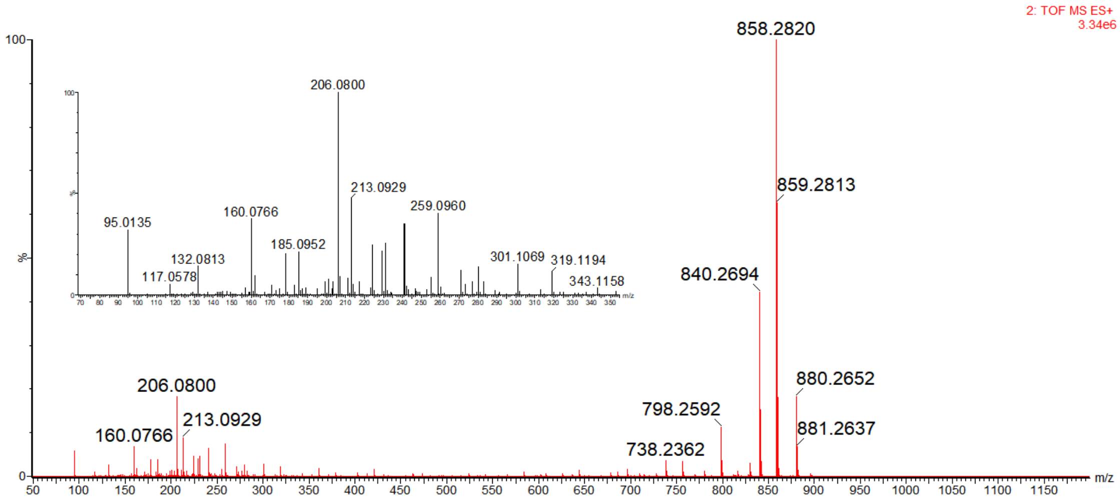

Figure S52. HRMS spectrum of compound **10**

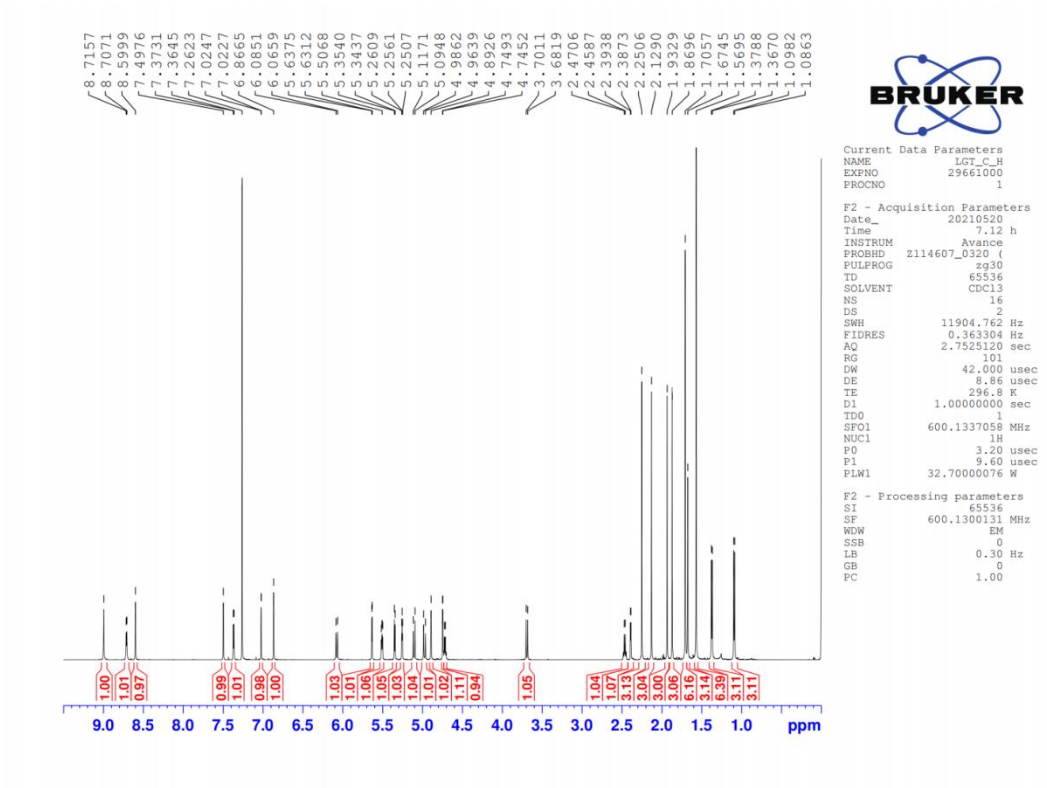

Figure S53. ^{1}H-NMR spectrum of compound **10**

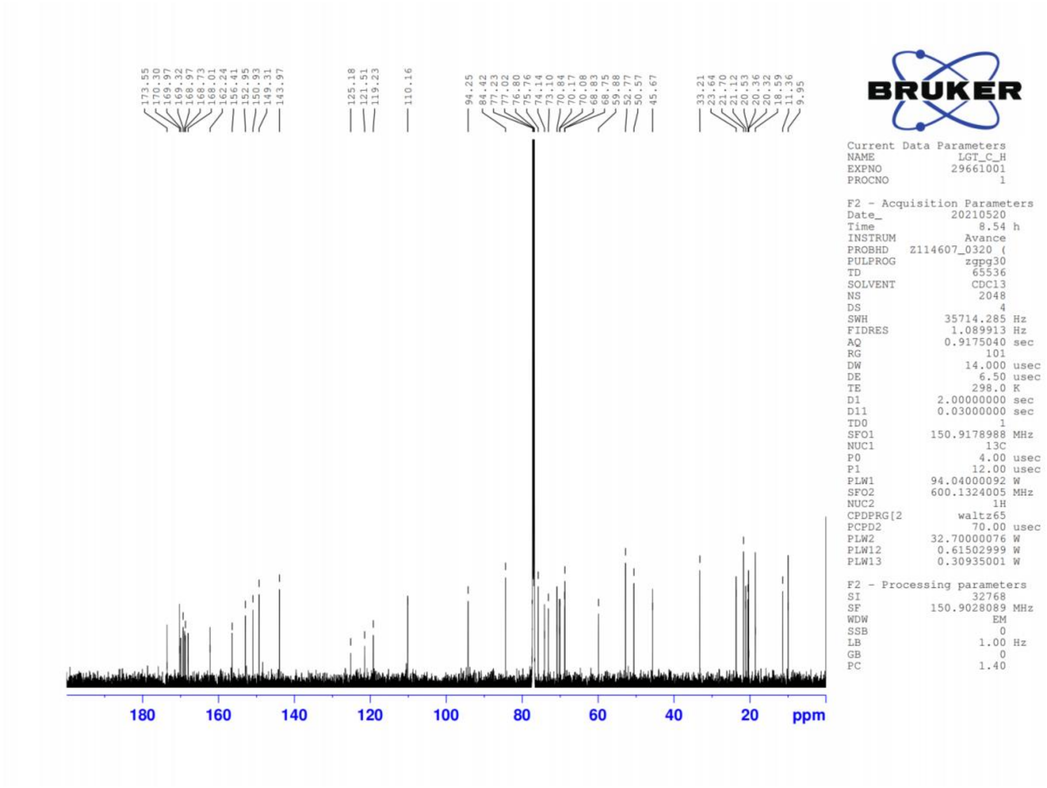

Figure S54. ^{13}C-NMR spectrum of compound **10**

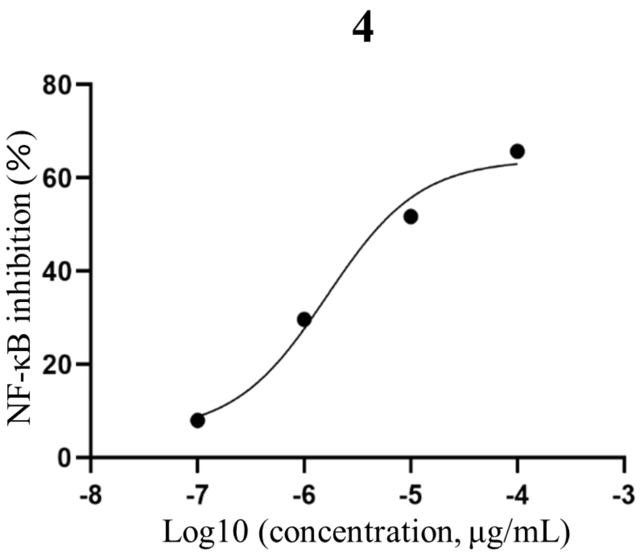

Figure S55. NF-κB inhibitory effect of compound **2**

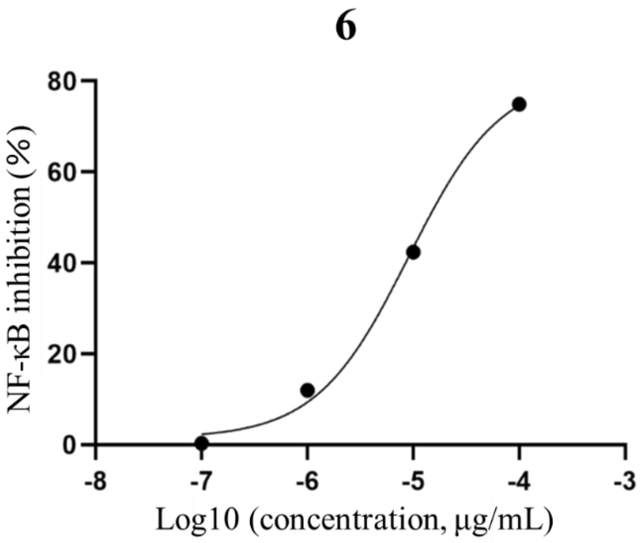

Figure S56. NF-κB inhibitory effect of compound **6**
